# Upper Bounds on the Colloid Separation Efficiency of Diffusiophoresis

**DOI:** 10.1021/acs.langmuir.5c06768

**Published:** 2026-05-07

**Authors:** Fernando Temprano-Coleto, Jeongmin Kim, Marcel M. Louis, Howard A. Stone

**Affiliations:** † Department of Mechanical and Aerospace Engineering, 6740 Princeton University, Princeton, New Jersey 08544, United States; ‡ Andlinger Center for Energy and the Environment, Princeton University, Princeton, New Jersey 08544, United States

## Abstract

The separation of colloidal particles from fluids is essential to ensure a safe global supply of drinking water, yet in the case of microscopic particles, it remains a highly energy-intensive process when using traditional filtration methods. Water cleaning through diffusiophoresis, spontaneous colloid migration in chemical gradients, effectively circumvents the need for physical filters, representing a promising alternative. This separation process is typically realized in internal flows, where a cross-channel electrolyte gradient drives particle accumulation at walls, with colloid separation slowly increasing in the streamwise direction. However, the maximum separation efficiency, achieved sufficiently downstream as diffusiophoretic migration (driving particle accumulation) is balanced by Brownian motion (inducing diffusive spreading), has not yet been characterized. In this work, we develop an asymptotic theory to predict colloid separation in this limit, deriving expressions for the water recovery, defined as the fraction of clean water that can be obtained from the suspension. We find that the mechanism by which the chemical permeates in the channel and the reaction kinetics governing its dissociation into ions play key roles in the process. Moreover, we identify four distinct regimes in which separation is controlled by different scaling laws involving Damköhler and Péclet numbers, which measure the ratios of reaction kinetics to ion diffusion and diffusiophoresis to Brownian motion, respectively. We also confirm the scaling of one of these regimes using microfluidic experiments where separation is driven by CO_2_ gradients. Our results shed light on pathways toward new, more efficient separations and are also applicable to quantify colloidal accumulation in the presence of chemical gradients in more general situations.

## Graphical abstract

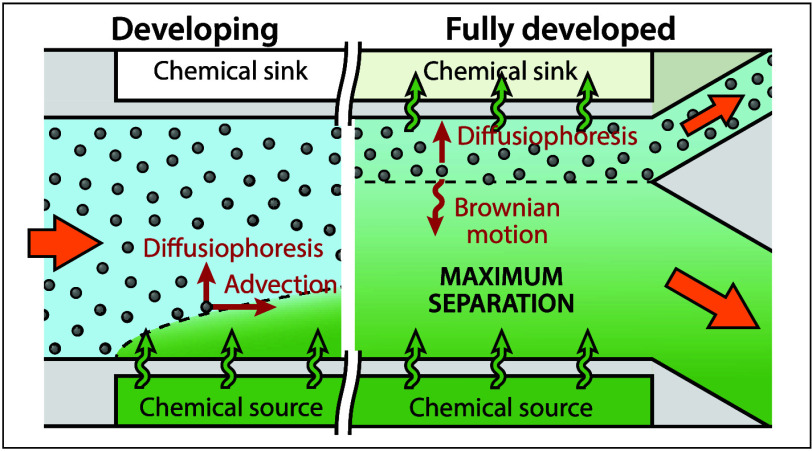

## Introduction

Colloidal suspensions spontaneously migrate in the presence of electrolyte gradients through a physicochemical mechanism known as diffusiophoresis. Since its discovery by Derjaguin^1,2^ and its subsequent theoretical formalization,^3–6^ many studies have used this phenomenon to manipulate particle motion at small scales. Applications include colloid focusing,^7–10^ custom particle migration,^11–13^ particle delivery into porous structures,^14–17^ or the characterization of colloidal properties,^18,19^ among others.^20,21^

Diffusiophoresis has also been shown to induce the continuous removal of colloidal particles from water.^22–27^ In this scenario, separation is typically achieved by field-flow fractionation, where a flowing colloidal suspension is subject to a cross-channel chemical gradient. The resulting phoretic motion drives particles to accumulate near channel walls, creating particle-depleted regions near the channel center that can be diverted to obtain clean water. This chemically driven process requires no electrical input beyond the power required for pumping, and circumvents the use of physical filters that become highly energy intensive for small particles since their membrane pore size must also scale with the particle dimensions.^28,29^ Therefore, diffusiophoresis has been highlighted as an energy-efficient alternative to traditional filtration, increasingly needed with the rise of emerging contaminants like microplastics and nanoplastics, now widespread in virtually every source of water.^30–32^

Existing theories to predict diffusiophoresis-driven colloid separation have so far focused on how particle distributions evolve downstream after a chemical cross-channel gradient is established^24,26^ (as in Figure 1a). However, the maximum degree of separation, which is achieved sufficiently downstream as the particle distributions become invariant in the streamwise direction (Figure 1b), has not yet been explored. Such a limit yields an upper bound of the fraction γ of clean water, often termed the “water recovery”, that can be obtained from a particle suspension through diffusiophoresis alone. Predicting this quantity, which we define precisely below, is in turn essential to design channel splittings (Figure 1) optimizing clean water output, and is therefore key to assess the potential scalability and feasibility of this type of separation.

**Figure 1:**
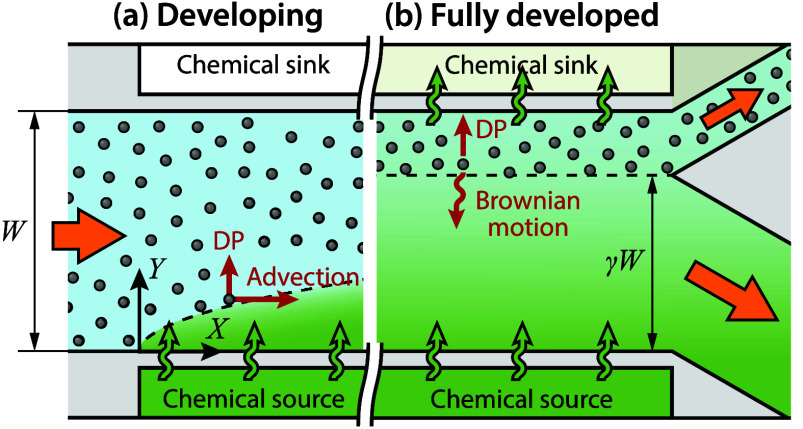
Colloid separation through diffusiophoresis in field-flow fractionation. (a) Upstream, the interplay between flow advection and the permeation of the chemical results in a developing distributions of chemical species and particles. (b) Sufficiently far downstream, all fields are fully developed and invariant in the streamwise direction, yielding the maximum fraction γ of clean water (the “water recovery”) that can be obtained through diffusiophoresis.

In this study, we formulate a continuum theory for this regime, where the two dominant effects are the diffusiophoresis-induced advection of particles, driving particles to accumulate at channel walls, and the Brownian motion of the colloids, which diffusively smooths the colloid distribution and hinders separation.

The main conclusion of our analysis is that separation depends strongly on the type of chemical source employed, distinguishing between a gas diffusing into the channel through a nonporous membrane and a liquid permeating through a porous membrane, two distinct configurations that have been realized in microfluidic experiments. Similarly, dissociation kinetics of the chemical into the ions driving the process also plays a key role, with different predictions depending on the rate of dissociation. Using asymptotic analysis, we provide scaling relations that predict separation in four distinct regimes, which are confirmed to be valid by numerical simulations. Finally, we provide experimental verification of one of these regimes by measuring colloid separation in microfluidic channels under a sustained gradient of carbon dioxide.

## Theory

### Problem Setup

We consider an aqueous suspension of solid particles flowing in a two-dimensional channel at steady state (Figure 1), with *X* and *Y* the streamwise and spanwise coordinates, respectively. The wall at *Y* = 0 is a membrane that allows a solute S to permeate into the channel from a source, and whose molar concentration we denote by $C_{s}\left(X,Y\right)≔\left[S\right]$ . This solute dissociates in water through the reaction

1 $$S\overset{k_{f}}{\underset{k_{r}}{⇌}}\nu_{C}C+\nu_{A}A$$

with C a cation, A an anion, $\nu_{C}$ and $\nu_{A}$ the number of cation and anion moles produced by the dissociation of one mole of S , and *k*_f_ and *k*_r_ the forward and reverse rate constants, respectively. Typically, the reverse reaction in eq 1 is neglected for strong electrolytes (e.g., salts like NaCl, KCl, CaCl_2_, ...), which are considered fully dissociated. However, in weak electrolytes like CO_2_, which has been used in experiments involving separation,^22,24,26^ both reactions are non-negligible and, as we show below, considering the full dissociation chemistry is key to make quantitative predictions. We further assume that eq 1 is the dominant equilibrium in the bulk liquid, thus regarding [ C ], [ A ] and [S] as much larger than the concentrations of any other species possibly arising from additional chemical reactions.

This assumption implies that we can consider the solution a binary electrolyte, in which case we can define a single “reduced” ionic concentration

2 $$C_{i}\left(X,Y\right)≔\frac{\left[C\right]}{\nu_{C}}=\frac{\left[A\right]}{\nu_{A}}$$

This simplification arises from the electroneutrality of the solution,^33^ which should only break down within distances of the order of the Debye–Hückel screening length λ_D_ of the (charged) channel walls. This length typically ranges between a few nanometers and a few micrometers, and is therefore much smaller than the width *W* of the channel in this work, i.e., λ_D_ ≪ *W*. We also consider the reactions in eq 1 to be elementary and the two species *C*_*s*_ and *C*_*i*_ to be sufficiently dilute to regard the electrolyte as an ideal solution. Accordingly, the reaction rates depend on concentration following the law of mass action, so we define $R≔k_{f}\left[S\right]-k_{r}{\left[C\right]}^{\nu_{C}}{\left[A\right]}^{\nu_{A}}$ as an overall chemical reaction rate. Using eq 2, we obtain

3 $$R\left(C_{s},C_{i}\right)=k_{f}C_{s}-k_{r}\nu_{C}^{\nu_{C}}\nu_{A}^{\nu_{A}}C_{i}^{\nu }$$

where we have defined $\nu ≔\nu_{C}+\nu_{A}$ . In the case of symmetric *z*:*z* electrolytes, such as those arising from the dissociation of salts like NaCl^34^ or gases like CO_2_,^20,22,24,26,35,36^ we have $\nu_{C}=\nu_{A}=1$ and ν = 2. However, the value of ν can be higher for asymmetric electrolytes such as CaCl_2_ (ν = 3), Na_3_PO_4_ (ν = 4), or Al_2_(SO_4_)_3_ (ν = 5), so we assume ν ≥ 2 in general.

The spatial distributions of *C*_*s*_ and *C*_*i*_ are therefore governed by two coupled partial differential equations

4a $$U\frac{\partial C_{s}}{\partial X}=D_{s}\left(\frac{\partial^{2}C_{s}}{\partial X^{2}}+\frac{\partial^{2}C_{s}}{\partial Y^{2}}\right)-R\left(C_{s},C_{i}\right)$$

4b $$U\frac{\partial C_{i}}{\partial X}=D_{i}\left(\frac{\partial^{2}C_{i}}{\partial X^{2}}+\frac{\partial^{2}C_{i}}{\partial Y^{2}}\right)+R\left(C_{s},C_{i}\right)$$

where *U*(*Y*) is the velocity field inside the channel, *D*_s_ is the diffusivity of the solute, and *D*_*i*_ is the ambipolar diffusivity^33^ of the ions. The system of eqs 4a and 4b gives rise to spatial gradients in the ionic concentration, which in turn induces a diffusiophoresis-driven migration velocity

5 $$U_{p}=\Gamma_{p}\frac{\nabla C_{i}}{C_{i}}$$

of the particles in the suspension.^5^ The constant Γ_p_ is the diffusiophoretic mobility, dependent on both the composition of the electrolyte and the properties of the particles, and which can be both positive (leading to “chemo-attracted” particles) or negative (resulting in “chemo-repelled” particles). We note that eq 5, while derived in the limit of vanishing double layers compared to the particle size (λ_D_/*R*_p_ → 0, with *R*_p_ the particle radius), admits corrections to Γ_p_ accounting for finite double layer thickness.^14,37^ This is especially important in cases where λ_D_ is comparable to the particle size λ_D_ ≲ *R*_p_, which can occur for sufficiently small ionic concentrations and particle sizes. In addition, eq 5 is valid for both valence-symmetric and asymmetric binary electrolytes.^38^ Additional ionic species beyond one cation and one anion preclude the definition of a single “reduced” concentration *C*_*i*_ and would lead to more complicated expressions for ***U***_p_ that lie outside the scope of our analysis.

Denoting the *X* and *Y* components of ***U***_p_, respectively, as *U*_p_ and *V*_p_, the concentration *N*(*X*, *Y*) of particles is given by

6 $$\frac{\partial \left(\left(U+U_{p}\right)N\right)}{\partial X}+\frac{\partial \left(V_{p}N\right)}{\partial Y}=D_{p}\left(\frac{\partial^{2}N}{\partial X^{2}}+\frac{\partial^{2}N}{\partial Y^{2}}\right)$$

where *D*_p_ is the diffusivity of the particles arising from their Brownian motion. After the chemical source is set in contact with the channel at *X* = 0 (Figure 1a), the solute, ions, and particles are initially developing, with *C*_*s*_, *C*_*i*_, and *N* slowly changing in the streamwise direction *X*. This case, as well as its implications for the separation of colloids, has been studied previously by several authors.^22–24,26^

In contrast, here we study the limit in which the system given by eqs 4a, 4b, and 6 reaches a fully developed state (Figure 1b). Sufficiently far downstream, the fields *C*_*s*_, *C*_*i*_ and *N* become invariant in the streamwise direction, i.e., *∂*_*X*_ *C*_*s*_ = *∂*_*X*_ *C*_*i*_ = 0, leading to

7a $$D_{s}\frac{d^{2}C_{s}}{dY^{2}}=\left[k_{f}C_{s}-k_{r}\nu_{C}^{\nu_{C}}\nu_{A}^{\nu_{A}}C_{i}^{\nu }\right]$$

7b $$D_{i}\frac{d^{2}C_{i}}{dY^{2}}=-\left[k_{f}C_{s}-k_{r}\nu_{C}^{\nu_{C}}\nu_{A}^{\nu_{A}}C_{i}^{\nu }\right]$$

In this fully developed regime, chemical gradients are sustained through the continuous diffusion of solute into the channel from the chemical source at *Y* = 0, but also through its withdrawal into a sink at *Y* = *W* (see Figure 1b). Accordingly, we set boundary conditions

8a $$C_{s}=C_{s0},\mathrm{at}Y=0$$

and

8b $$C_{s}=C_{s*},\mathrm{at}Y=W$$

where *C*_*s*0_ and *C*_s*_ are reference solute concentrations imposed by the chemical source and sink, respectively.

We can then estimate the typical ionic concentrations in the channel as the values *C*_*i*0_ and *C*_i*_ set by *C*_*s*0_ and *C*_s*_, respectively, at chemical equilibrium. These values can be obtained imposing detailed balance in the reaction term *R*(*C*_s*_, *C*_i*_) = *R*(*C*_*s*0_, *C*_*i*0_) = 0, yielding

9a $$C_{i0}={\left(\frac{k_{f}C_{s0}}{k_{r}\nu_{C}^{\nu_{C}}\nu_{A}^{\nu_{A}}}\right)}^{1/\nu }$$

9b $$C_{i*}={\left(\frac{k_{f}C_{s*}}{k_{r}\nu_{C}^{\nu_{C}}\nu_{A}^{\nu_{A}}}\right)}^{1/\nu }$$

Boundary conditions for the ionic species *C*_*i*_ depend on the mechanism of solute transport through the channel membrane walls. On one hand, for a liquid reservoir the solute itself permeates into the channel via fluid flow through the porous wall. This setup can be achieved, for instance, using hydrogel membranes in microfluidics.^8,34,39–41^ In this case, the solution composition at walls must match the equilibrium ionic concentrations of source and sink, i.e.

10a $$C_{i}=C_{i0},\mathrm{at}Y=0$$

and

10b $$C_{i}=C_{i*},\mathrm{at}Y=W$$

On the other hand, in gas sources the chemical permeates through the membrane by way of a solution-diffusion mechanism,^42^ then dissolves in the aqueous solution to produce the solute, which in turn dissociates into the ions. This setup has been realized in microfluidics using CO_2_ and gas-permeable polydimethylsiloxane (PDMS) devices.^22,24,26^ Since the walls are only gas-permeable, ions in solution cannot diffuse through them, such that

11 $$\frac{dC_{i}}{dY}=0,,\mathrm{at}Y=0\mathrm{and}Y=W$$

Regarding the concentration of particles, in the fully developed regime we expect that *∂*_*X*_ *N* = 0, leading to

12 $$\frac{d\left(V_{p}N\right)}{dY}=D_{p}\frac{d^{2}N}{dY^{2}}$$

which is supplemented by boundary conditions specifying a zero particle flux at channel walls

13 $$V_{p}N-D_{p}\frac{dN}{dY}=0,,\mathrm{at}Y=0\mathrm{and}Y=W$$

Intuitively, eq 13 ensures that any particle flux *V*_p_ *N* originating from diffusiophoretic migration is compensated by Brownian diffusion −*D*_p_ *∂*_*Y*_ *N* to yield no net particle flux through either wall. Since the particle concentration given by eqs 12 and 13 is only defined up to a multiplicative constant, we also fix an average value *N*_0_ given by

14 $$\frac{1}{W}\int_{0}^{W}N\left(Y\right)dY=N_{0}$$

### Nondimensionalization

We seek to understand how the problem parameters affect the spatial distribution of particles *N*(*Y*), so we first define the nondimensional variables *y* ≔ *Y*/*W*, *c*_*s*_ ≔ *C*_*s*_/*C*_*s*0_, *c*_*i*_ ≔ *C*_*i*_/*C*_*i*0_, and *n* ≔ *N*/*N*_0_. Following this normalization, the particle concentration follows an ordinary differential equation (ODE) given by

15 $$\pm {Pe}_{p}\frac{d}{dy}\left(v_{p}n_{\pm }\right)=\frac{d^{2}n_{\pm }}{dy^{2}}$$

where we define the particle Péclet number as

16 $${Pe}_{p}≔\frac{\left|\Gamma_{p}\right|}{D_{p}}>0$$

and where the signs distinguish between the case where the mobility is positive Γ_p_ > 0 and the particles *n*_+_(*y*) are chemo-attracted, from the case where Γ_p_ < 0 and the particles *n*_–_(*y*) are chemo-repelled. We have also defined a nondimensional particle migration velocity *v*_p_ ≔ *V*_p_ *W*/Γ_p_, related to *c*_*i*_(*y*) through

17 $$v_{p}=\frac{1}{c_{i}}\frac{dc_{i}}{dy}$$

Equation 15 along with eq 17 and conditions

18a $$\pm {Pe}_{p}v_{p}n_{\pm }-\frac{\partial n_{\pm }}{\partial y}=0,\mathrm{at}y=0\mathrm{and}y=1$$

18b $$\int_{0}^{1}n_{\pm }\left(y\right)dy=1$$

which are the nondimensional equivalents of eqs 13 and 14, respectively, can be integrated for an arbitrary ionic concentration *c*_*i*_(*y*). The solution is

19 $$n_{\pm }\left(y\right)=\frac{c_{i}{\left(y\right)}^{\pm {Pe}_{p}}}{\int_{0}^{1}c_{i}{\left(y\right)}^{\pm {Pe}_{p}}dy}$$

This power-law behavior of the particle distribution with exponent *Pe*_p_ has also been recently reported in theories for colloid trapping in chemical gradients.^10^ While eq 19 is an exact result, the particles *n*_±_(*y*) still depend on the concentration *c*_*i*_(*y*), which is in turn given by the dimensionless versions of eq 7a and 7b, namely

20a $$\frac{1}{Da_{s}}\frac{d^{2}c_{s}}{dy^{2}}=\left[c_{s}-c_{i}^{\nu }\right]$$

20b $$\frac{1}{Da_{i}}\frac{d^{2}c_{i}}{dy^{2}}=-\left[c_{s}-c_{i}^{\nu }\right]$$

Here, we have defined two Damköhler numbers

21a $${Da}_{s}≔\frac{k_{f}W^{2}}{D_{s}}$$

21b $$Da_{i}:=\frac{k_{f}W^{2}C_{s0}}{D_{i}C_{i0}}$$

which indicate the relative rates of the chemical reactions given by eq 1 with respect to diffusion.

The coupled eqs 20a and 20b can be simplified, first noting that adding eqs 20a and 20b leads to

22 $$\frac{1}{Da_{s}}\frac{d^{2}c_{s}}{dy^{2}}+\frac{1}{Da_{i}}\frac{d^{2}c_{i}}{dy^{2}}=0$$

We can then differentiate eq 20b twice and combine it with eq 22 to obtain

23 $$\frac{d^{4}c_{i}}{dy^{4}}=Da_{i}\frac{d^{2}\left(c_{i}^{\nu }\right)}{dy^{2}}+Da_{s}\frac{d^{2}c_{i}}{dy^{2}}$$

thereby reducing the coupled system to a single fourth-order nonlinear equation for *c*_*i*_, whose solution requires four boundary conditions to be determined uniquely.

In the case of liquid sources, the boundary conditions are the nondimensional versions of eqs 8a-b and eqs 10a-b for the solute and ions, respectively. Combining those expressions with eq 20b, it is possible to cast the four boundary conditions in terms of *c*_*i*_ only, leading to

24a $$c_{i}=1,\mathrm{at}y=0$$

$$c_{i}=\varepsilon^{1/\nu },\mathrm{at}y=1$$ 24b

24c $$\frac{d^{2}c_{i}}{dy^{2}}=0,\mathrm{at}y=0\mathrm{and}y=1$$

where we define $\varepsilon ≔C_{s*}/C_{s0}={\left(C_{i*}/C_{i0}\right)}^{\nu }$ as the ratio of solute concentration between the sink and the source. Since, in practice, the sink could be engineered to have a very low concentration such that ε ≪ 1, taking ε = 0 would seem as a natural simplification. However, in the particular case of liquid sources, a “perfect” sink with ε = 0 would lead to a zero concentration *c*_*i*_(1) = 0 at the wall and, as a consequence, to a divergent particle velocity |*v*_p_| → *∞*, according to eq 17. This would in turn result in an unbounded distribution *n*_–_(*y*) of particles in the case in which they are chemo-repelled, highlighting that a small (but nonzero) value of ε has an effect that is not small in the case of a liquid source. While it is possible to regularize this divergence including other physical effects like a concentration-dependent mobility,^37^ here we will simply assume a nonzero value 0 < ε < 1 in the case of a liquid source.

In the case of gas sources, the boundary conditions are the nondimensional versions of eqs 8a, 8b, and 11, which can once again be combined with eq 20b to arrive at

25a $$c_{i}^{\nu }-\frac{1}{Da_{i}}\frac{d^{2}c_{i}}{dy^{2}}=1,\mathrm{at}y=0$$

25b $$c_{i}^{\nu }-\frac{1}{Da_{i}}\frac{d^{2}c_{i}}{dy^{2}}=0,\mathrm{at}y=1$$

25c $$\frac{dc_{i}}{dy}=0,\mathrm{at}y=0\mathrm{and}y=1$$

where we have taken ε = 0 since, in this case, the limit does not lead to a singular particle velocity because the no-flux condition (eq 25c) prevents *c*_*i*_ from becoming zero at the sink. This means that imperfect sinks with small values 0 < ε ≪ 1 have only small, higher order effects on the particle distribution in the case of gas sources.

Neither the boundary-value problem for liquid sources, given by eqs 23 and 24a–c, nor the one for gas sources, given by eqs 23 and 25a–c, are exactly solvable in general. However, we can obtain key insights about the structure of their solutions from an asymptotic analysis^43–45^ of the problem in practically relevant physical regimes. To that end, we first estimate the typical ranges of values of the three dimensionless numbers (*Pe*_p_, *Da*_*s*_, and *Da*_*i*_) to identify different distinguished limits in the parameter space.

### Estimation of Parameters

In order to estimate the Péclet number *Pe*_p_, the particle diffusivity *D*_p_ can be calculated using the Stokes–Einstein relation *D*_p_ = *k*_B_ *T*/(6πμ*R*_p_), with *k*_B_ the Boltzmann constant, *T* the absolute temperature, μ the dynamic viscosity of the medium, and *R*_p_ the particle radius. This formula neglects electrostatic effects and is strictly valid only in the limit of a thin double layer compared to the particle size λ_D_ ≪ *R*_p_, but is nevertheless useful as an order-of-magnitude estimate. For water at room temperature, and for particle sizes in the range *R*_p_ = *O*(10 nm–10 μm) relevant in practice, we have diffusivities *D*_p_ = *O*(10^–11^–10^–14^ m^2^ s^–1^). In turn, at moderate concentrations *C*_*i*0_ = *O*(0.1–10 mM) we can expect diffusiophoretic mobilities Γ_p_ to be of the same order of magnitude as the ambipolar diffusivity of the electrolyte.^37^ Most small ions in aqueous solutions have *D*_*i*_ = *O*(10^–10^–10^–9^ m^2^ s^–1^), so we expect that *Pe*_p_ = |Γ_p_|/*D*_p_ = *O*(10^2^–10^4^ ). Therefore, we only consider the relevant asymptotic limit of *Pe*_p_ ≫ 1. This range of values of *Pe*_p_ should not come as a surprise, since diffusiophoresis is sought in applications precisely because it overcomes Brownian motion, inducing the directed migration of colloids.^7,14,16^

Estimating the values of the two Damköhler numbers is more challenging due to the wide range of variation of the kinetic constants *k*_f_ and *k*_r_. Nevertheless, we can distinguish qualitatively between two limiting key regimes, given that the ratio of Damköhler numbers (eqs 21a-b) yields

26 $$\frac{Da_{i}}{Da_{s}}=\frac{D_{s}}{D_{i}}\frac{C_{s0}}{C_{i0}}$$

Since we can expect *D*_*s*_ and *D*_*i*_ to be of the same order of magnitude, the quantity *Da*_*i*_/*Da*_*s*_ essentially indicates how displaced is the chemical equilibrium given by eq 1. If *Da*_*i*_ ≪ *Da*_*s*_, then ionic concentrations are dominant, *C*_*i*0_ ≫ *C*_*s*0_, and the dissociation is strong. Conversely, if *Da*_*i*_ ≫ *Da*_*s*_, then *C*_*i*0_ ≪ *C*_*s*0_ and the dissociation is weak. For instance, common salts are typically regarded as strong electrolytes, and while their association reaction is typically neglected, some studies report very small values of their equilibrium constants, defined as $K_{\mathrm{assoc}}=C_{s0}/{C_{i0}}^{2}$ . For example, for NaCl and KCl, *K*_assoc_ = *O*(10^–4^ m^3^/mol),^46^ leading to $Da_{i}/Da_{s}\approx \sqrt{K_{\mathrm{assoc}}C_{s0}}=O$ ­(10^–2^) at solute concentrations of *C*_*s*0_ = *O*(1 mM). On the other hand, weak electrolytes like CO_2_ or NH_3_ have dissociation constants $K_{\mathrm{dissoc}}={C_{i0}}^{2}/C_{s0}$ with values *K*_dissoc_ = 4.3 × 10^–4^ mM and *K*_dissoc_ = 5.6 × 10^–7^ mM, respectively,^47^ which lead to $Da_{i}/Da_{s}=\sqrt{C_{s0}/K_{\mathrm{dissoc}}}$ with values of *Da*_*i*_/*Da*_*s*_ ≈ 48 and *Da*_*i*_/*Da*_*s*_ ≈ 1.3 × 10^3^, respectively, at solute concentrations of *C*_*s*0_ = *O*(1 mM).

Figure 2 displays the numerical solution of the problem (eq 23) for ν = 2, *Pe*_p_ ≫ 1, in both the weak and strong dissociation limits, and using either boundary conditions given byeqs 24a–c for a liquid source (Figure 2a–c) or by eqs 25a–c for a gas source (Figure 2b–d). We use *Da*_*s*_ = 1 in all cases, *Da*_*i*_ = 50 for weak dissociation, and *Da*_*i*_ = 1/50 for strong dissociation, which are chosen as a representative set of values to capture each regime qualitatively. Additionally, we pick ε = 0.1 to regularize the cases with a liquid source and ε = 0 for gas sources. Note that the concentration and particles have noticeably different spatial distributions depending on the boundary conditions and the regime of dissociation.

**Figure 2:**
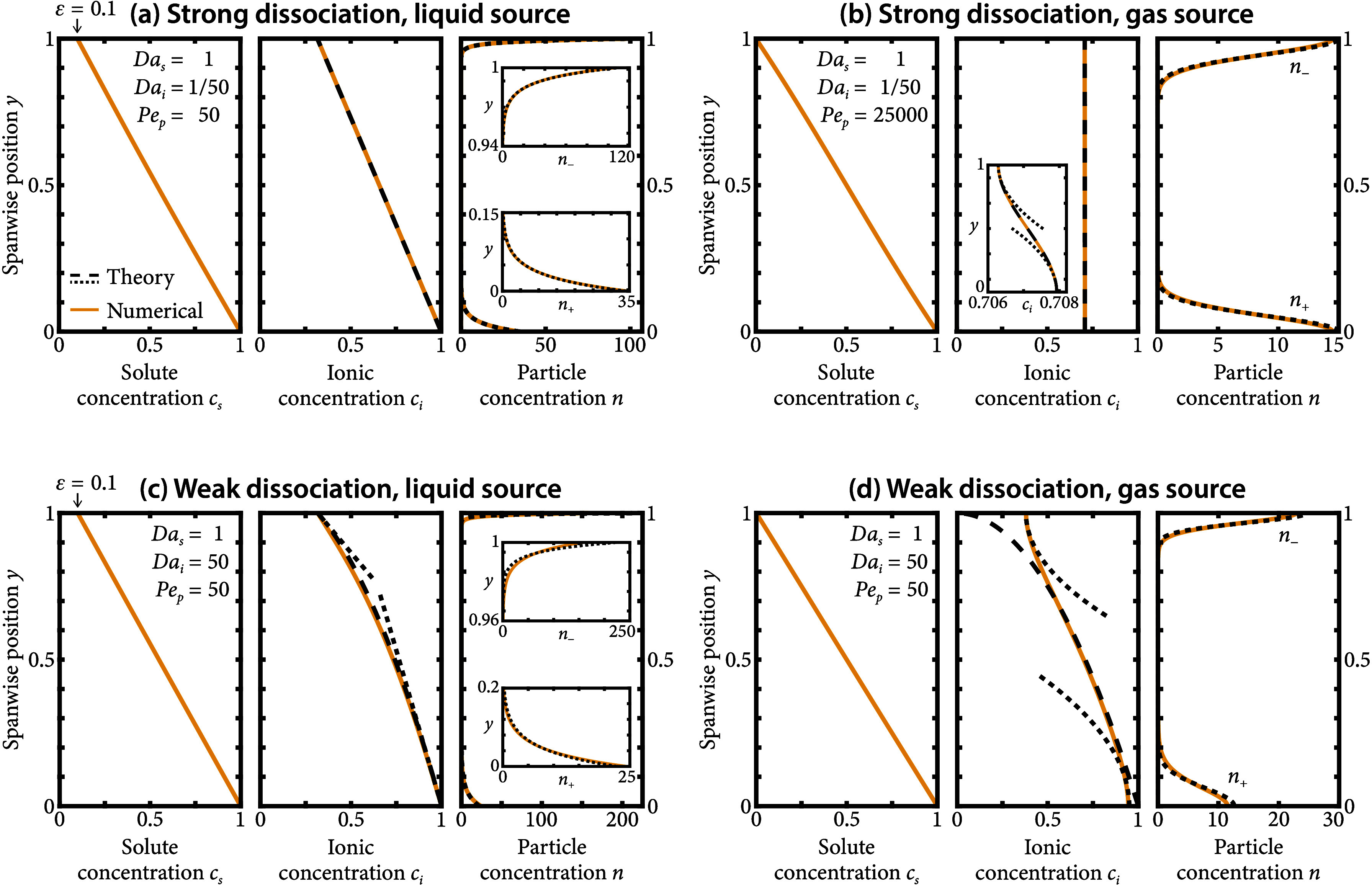
Colloid separation through diffusiophoresis strongly depends on the dissociation chemistry and permeating mechanism of the chemical. Each subfigure represents one of the four identified characteristic regimes, namely: (a) strong dissociation [*Da*_*i*_ ≪ *Da*_*s*_] with a liquid source (eqs 24a–c), (b) strong dissociation [*Da*_*i*_ ≪ *Da*_*s*_] with a gas source (eqs 25a–c), (c) weak dissociation [*Da*_*i*_ ≫ *Da*_*s*_] with a liquid source (eqs 24a–c), and (d) weak dissociation [*Da*_*i*_ ≫ *Da*_*s*_] with a gas source (eqs 25a–c). All solid lines represent numerical solutions of the full model given by eqs 19 and 23, subject to boundary conditions given by either eqs 24a–c or eqs 25a–c. All dashed lines correspond to asymptotic solutions that are either globally valid or “outer” solutions valid only away from walls. All dotted lines correspond to local approximations near walls, obtained either by local expansion of the globally valid solutions or as “inner” solutions by asymptotic matching. All insets are magnifications of the solutions for a clearer visualization of their spatial structure.

In the next three subsections, we analyze the asymptotic structure of the problem to obtain quantitative predictions for the separation efficiency. We seek to obtain the simplest possible leading-order expression for the particle distributions *n*_±_(*y*) as a function of *Pe*_p_, *Da*_*i*_, *Da*_*s*_ (and, in the case of liquid sources, also ε), neglecting all higher order effects.

### Particle Distributions at *Pe*_p_ ≫ 1

We first analyze the exact solutions for the distribution of particles given by eq 19, in the relevant case of *Pe*_p_ ≫ 1. In this limit, we can expect *n*_±_(*y*) to have a boundary layer structure,^43,44^ with particles accumulating in thin regions near walls. We denote the width of these regions as δ_+_, for the chemo-attracted particles accumulating at the chemical source, and δ_–_, for the chemo-repelled particles accumulating at the chemical sink. For high enough Péclet number, these regions can get thin enough to approximate the ionic concentration *c*_*i*_(*y*) by its leading-order Taylor expansion, since deviations only become noticeable outside the boundary layers δ_±_. We first introduce local coordinates

27a $$y_{+}=y$$

27b $$y_{-}=1-y$$

to use a more compact notation that ensures *y*_±_ = 0 at either wall, and then pose a linear expansion

28 $$c_{i}\left(y_{\pm }\right)=c_{i}\left(0\right)+\frac{dc_{i}}{dy_{\pm }}\left(0\right)y_{\pm }+O\left({y_{\pm }}^{2}\right)$$

in the case of a liquid source, and a quadratic expansion

$$c_{i}\left(y_{\pm }\right)=c_{i}\left(0\right)+\frac{d^{2}c_{i}}{dy_{\pm }^{2}}\left(0\right)\frac{{y_{\pm }}^{2}}{2}+O\left({y_{\pm }}^{3}\right)$$ 29

for gas sources, since in that case we have no ionic flux at walls as given by eq 25c. Introducing these expansions into the solution (eq 19) for the particles, and after an asymptotic analysis that we detail in the Supporting Information, we can show that, neglecting all exponentially small terms, the leading-order approximations *n* ^(0)^ for the particles are

30a $$n_{\pm }^{\left(0\right)}\left(y_{\pm }\right)=\frac{e^{-y_{\pm }/\delta_{\pm }}}{\delta_{\pm }}\mathrm{for liquid sources}$$

and

30b $$n_{\pm }^{\left(0\right)}\left(y_{\pm }\right)=\frac{2e^{-{\left(\frac{y_{\pm }}{\delta_{\pm }}\right)}^{2}}}{\sqrt{\pi }\delta_{\pm }}\mathrm{for gas sources}$$

The analysis also allows us to identify and define the widths δ_±_ of the particle boundary layers, which we obtain as

31a $$\delta_{\pm }=\frac{c_{i}\left(0\right)}{\left|\frac{dc_{i}}{dy_{\pm }}\left(0\right)\right|}{{Pe}_{p}}^{-1}\mathrm{for liquid sources}$$

$$\delta_{\pm }={\left[\frac{2c_{i}\left(0\right)}{\left|\frac{d^{2}c_{i}}{dy_{\pm }^{2}}\left(0\right)\right|}\right]}^{1/2}{{Pe}_{p}}^{-1/2}\mathrm{for gas sources}$$ 31b

where we emphasize that the values of *c*_*i*_ and its derivative are evaluated at walls *y*_±_ = 0 or, equivalently, at either *y* = 0 (for the “+” case) or *y* = 1 (for “–”).

In summary, the particle distributions in the limit of large particle Péclet number *Pe*_p_ ≫ 1 will tend to either exponential (eq 30a) or Gaussian (eq 30b) curves near channel walls, depending on whether the chemical source delivers a liquid or a gas. Furthermore, these distributions and, by extension, the separation of colloids, is dictated by a single boundary layer parameter δ_±_ at each wall, which itself depends on both the relative importance of diffusiophoresis versus Brownian motion (through *Pe*_*s*_) and potentially on the dissociation chemistry (through the ionic concentration profile *c*_*i*_), as highlighted by eqs 31a and 31b. In the next two subsections, we evaluate *c*_*i*_(*y*) and their their relevant expansions near walls to obtain δ_±_ using eqs 31a and 31b.

### Strong Dissociation (*Da*_*i*_ ≪ *Da*_*s*_)

Here, we assume an order-one value of *Da*_*s*_ = *O*(1), while taking a small *Da*_*i*_ ≪ 1 such that *Da*_*i*_/*Da*_*s*_ ≪ 1. Accordingly, we expand *c*_*i*_ in powers of *Da*_*i*_ as

32 $$c_{i}=c_{i}^{\left(0\right)}+Da_{i}c_{i}^{\left(1\right)}+O\left({{Da}_{i}}^{2}\right)$$

in the governing eq 23. At leading-order, we obtain a linear ODE

$$\frac{d^{4}c_{i}^{\left(0\right)}}{{dy}^{4}}=Da_{s}\frac{d^{2}c_{i}^{\left(0\right)}}{{dy}^{2}}$$ 33

that results in a different ionic profile $c_{i}^{\left(0\right)}\left(y\right)$ depending on the specific boundary conditions, as we show next.

#### Liquid Sources

Solving eq 33 subject to boundary conditions (eqs 24a–c) results in a linear profile

34 $$c_{i}^{\left(0\right)}\left(y\right)=1-\left(1{-\varepsilon }^{1/\nu }\right)y$$

which, as displayed in the central panel of Figure 2a, is indistinguishable from the full numerical solution of eq 23. We can then easily obtain the Taylor expansions *c*_*i*_(*y*_+_) = 1 – (1 – ε^1/ν^)*y*_+_ and *c*_*i*_(*y*_–_) = ε^1/ν^ + (1 – ε^1/ν^)*y*_–_ at both walls which, when introduced in eqs 31a and 31b, lead to

35a $$\delta_{+}=\frac{1}{1-\varepsilon^{1/\nu }}{{Pe}_{p}}^{-1}$$

35b $$\delta_{-}=\frac{\varepsilon^{1/\nu }}{\left(1-\varepsilon^{1/\nu }\right)}{{Pe}_{p}}^{-1}$$

The solutions resulting from using eqs 35a-b in the profiles given by eqs 30a-b are plotted in the right panel of Figure 2a, showing good agreement with the numerical results. The formulas given by eqs 35a-b highlight the boundary layer structure, with widths δ_+_ ≪ 1 and δ_–_≪ 1 that are small since *Pe*_p_ ≫ 1 and ε < 1, which can be confirmed graphically in Figure 2a. This spatial distribution is precisely what is sought to optimize the efficiency of separation and maximize the output of clean water (the filtrate) with respect to the total influx of contaminated water (the feed). In the case of chemo-repelled particles, accumulation is enhanced due to the lower ionic concentration *c*_*i*_(1) at the chemical sink, which in turn leads to a higher diffusiophoretic velocity. This is apparent from the fact that the expressions for the boundary layer thicknesses (eqs 35a and 35b) are identical except for an extra factor of ε^1/ν^ < 1, making δ_–_ < δ_+_ and the chemo-repelled particles accumulate more strongly at the top (*y* = 1) wall, as shown by Figure 2a. In fact, as ε → 0 and the membrane becomes a perfect sink, the boundary layer thickness δ_–_ tends to zero and the particle distribution becomes singular, emphasizing the strong effect of ε. Note also that in this particular case the separation does not depend on the reaction chemistry at leading order, given that δ_+_ and δ_–_ do not depend on *Da*_*s*_ or *Da*_*i*_.

#### Gas Sources

We proceed by solving eq 33 with boundary conditions (eqs 25a–c) for gas sources. At leading order in *Da*_*i*_, the solution is $c_{i}^{\left(0\right)}\left(y\right)=A$ , with *A* some undetermined constant. This result highlights that this case is characterized by very mild gradients giving a constant ionic concentration and therefore a zero particle velocity at leading order. Physically, this can be understood by noting that the strong dissociation depletes the solute and drives the vast majority of the chemical into the ions which, owing to their no-flux conditions at both walls, tend to a flat profile. The numerical solution plotted in the inset of Figure 2b, however, reveals some very mild gradients, which can only be captured by solving for $c_{i}^{\left(1\right)}\left(y\right)$ in the first-order problem. A detailed calculation (see the Supporting Information) leads to

36a $$c_{i}^{\left(0\right)}\left(y\right)=2^{-1/\nu }$$

36b $$c_{i}^{\left(1\right)}\left(y\right)=-\frac{\left[y-\frac{1}{2}+\frac{e^{\sqrt{Da_{s}}\left(1-y\right)}-e^{\sqrt{Da_{s}}y}}{\sqrt{Da_{s}}\left(1+e^{\sqrt{Da_{s}}}\right)}\right]}{2{{Da}_{s}}^{1/2}\tanh\left(\sqrt{Da_{s}}/2\right)}$$

with the approximate first-order solution $c_{i}^{\left(0\right)}+Da_{i}c_{i}^{\left(1\right)}$ plotted with a dashed line in Figure 2b and showing excellent agreement with the numerical simulations. It is straightforward to obtain local expansions of *c*_*i*_ at walls, which are now parabolic and displayed with dotted lines in the inset of Figure 2b. Introducing these expansions in eqs 31a-b, we obtain

$$\delta_{+}=\delta_{-}=2^{\left(2\nu -1\right)/2\nu }{{Pe}_{p}}^{-1/2}{{Da}_{i}}^{-1/2}$$ 37

A closer look at eq 37 shows that particle distributions are symmetric and, since *Da*_*i*_ ≪ 1 in this case, the Péclet number must be very large for the boundary layers δ_+_ and δ_–_ to be small. In fact, a detailed analysis (see the Supporting Information) reveals that $Pe_{p}\gg {{Da}_{i}}^{-1}$ to have noticeable particle accumulation. Indeed, in Figure 2 all cases display noticeable particle accumulation for a moderately large *Pe*_p_ = 50 except for strong dissociation and gas sources, for which *Pe*_p_ = 25 000 to have accumulation comparable to the other three scenarios. This is, once again, a direct consequence of the weak gradients that appear in this case, which strongly limits the feasibility of the separation and suggests that gases with very fast dissociation reactions must be avoided in practice.

### Weak Dissociation (*Da*_*i*_ ≫ *Da*_*s*_)

We now keep an order-one value of *Da*_*s*_ = *O*(1) but take a large *Da*_*i*_ ≫ 1 such that *Da*_*i*_/*Da*_*s*_ ≫ 1. Accordingly, we pose an expansion

38 $$c_{i}=c_{i}^{\left(0\right)}+{{Da}_{i}}^{-1}c_{i}^{\left(1\right)}+O\left({{Da}_{i}}^{-2}\right)$$

in eq 23 which, at leading-order, yields

39 $$\frac{d^{2}}{dy^{2}}\left[{\left(c_{i}^{\left(0\right)}\right)}^{\nu }\right]=0$$

This equation can be integrated to obtain the solution $c_{i}^{\left(0\right)}={\left[K_{1}+K_{2}y\right]}^{1/\nu }$ , with *K*_1_ and *K*_2_ integration constants. Since, in general, one cannot impose the four required boundary conditions given by either eqs 24a–c or eqs 25a–c with only two constants, *c*_*i*_ also has a boundary layer structure. The leading-order eq 39 is therefore only an “outer” equation not valid sufficiently close to the walls, where other terms must become non-negligible to ensure all boundary conditions are satisfied. We tackle this boundary layer problem through the method of matched asymptotic expansions,^43–45^ with all the details available in the Supporting Information, and outline the results in the next two subsections.

#### Liquid Sources

In this case, two boundary layers of width $l_{+}=l_{-}=O\left({{Da}_{i}}^{-1/2}\right)$ form at each wall, with two corresponding “inner” linear ODEs that can be formulated in these regions. Note the contrast between the chemical boundary layers *l*_±_ for *c*_*i*_(*y*) and the particle boundary layers δ_±_ for *n*(*y*), which scale differently. The solution to these inner problems can be matched to the outer solution $c_{i}^{\left(0\right)}={\left[K_{1}+K_{2}y\right]}^{1/\nu }$ to obtain the constants, leading to $c_{i}^{\left(0\right)}={\left[1-\left(1-\varepsilon \right)y\right]}^{1/\nu }$ , which is displayed with dashed lines in Figure 2c. As in previous cases, we only seek to find local expansions of *c*_*i*_(*y*) near walls, obtaining

40a $$c_{i}\approx 1-\frac{1-\varepsilon }{\nu }y_{+}\mathrm{at the source}$$

$$c_{i}\approx \varepsilon^{1/\nu }+\frac{\varepsilon^{\left(1-\nu \right)/\nu }\left(1-\varepsilon \right)}{\nu }y_{-}\mathrm{at the sink}$$ 40b

which are plotted as dotted lines in Figure 2c. Introducing these expressions in eqs 31a-b, we obtain

41a $$\delta_{+}=\left(\frac{\nu }{1-\varepsilon }\right){{Pe}_{p}}^{-1}$$

41b $$\delta_{-}=\left(\frac{\nu \varepsilon }{1-\varepsilon }\right){{Pe}_{p}}^{-1}$$

These expressions depend on the reaction order (with wider layers for larger ν), but are still independent of kinetics since *Da*_*i*_ and *Da*_*s*_ do not appear in eqs 41a-b. As in the case of a strong dissociation with a liquid source, the boundary layer δ_–_ also vanishes as ε → 0, although in this case the effect of a small concentration at the sink is stronger given that δ_–_ depends on ε as opposed to ε^1/ν^. This is evident comparing the chemo-repelled particle distribution in Figure 2a and c, with the latter reaching larger particle accumulation near the sink wall.

#### Gas Sources

The last case involves a gas source and weak dissociation, with CO_2_-driven diffusiophoresis as a prominent example.^24,26,35,36^ Matched asymptotics reveals that the boundary layer at the source is governed by a linear inner equation with $l_{+}=O\left({{Da}_{i}}^{-1/2}\right)$ , while at the sink the inner equation is nonlinear and leads to $l_{-}=O\left({{Da}_{i}}^{-\nu /\left(3\nu -1\right)}\right)$ . The full analysis (see the Supporting Information) yields the local expansions

42a $$c_{i}\approx 1-\frac{{{Da}_{i}}^{-1/2}}{\nu^{3/2}}-\frac{{{Da}_{i}}^{1/2}}{2\nu^{1/2}}{y_{+}}^{2}\mathrm{at the source}$$

$$c_{i}\approx \alpha {{Da}_{i}}^{-1/(3\nu -1)}+\frac{\alpha^{\nu }}{2}{{Da}_{i}}^{\left(2\nu -1\right)/(3\nu -1)}{y_{-}}^{2}\mathrm{at the sink}$$ 42b

plotted with dotted lines in Figure 2d. The constant α cannot be calculated in closed form due to the nonlinear inner problem, but can be easily obtained by numerically solving the boundary-value problem

43a $$\frac{d^{2}f}{d\xi^{2}}=f^{\nu }-\xi$$

43b $$\frac{df}{d\xi }=0,\mathrm{at}\xi =0$$

43c $$f\to \xi^{1/\nu },\mathrm{as}\xi \to \infty$$

to then take α = *f*(0). Note that while the reaction order ν appears in eqs 43a–c, the constant α has a very weak dependence on ν, as shown in Table 1.

**Table 1:** Values of the Exponent −*ν*/(3*ν* – 1) and the Constant α (Obtained Numerically) Appearing in eqs 42b and 44b, as a Function of the Reaction Order *ν*

ν	2	3	4	5
$-\frac{\nu }{3\nu -1}$	–2/5	–3/8	–4/11	–5/14
α	0.830965	0.839455	0.855196	0.869162

Introducing the parabolic local expansions (eqs 42a-b) into eqs 31a-b leads to boundary layer widths given by

44a $$\delta_{+}=\left(2^{1/2}\nu^{1/4}\right){{Da}_{i}}^{-1/4}{{Pe}_{p}}^{-1/2}$$

$$\delta_{-}=\left(2^{1/2}\alpha^{\left(1-\nu \right)/2}\right){{Da}_{i}}^{-\nu /\left(3\nu -1\right)}{{Pe}_{p}}^{-1/2}$$ 44b

In this case, the boundary layers clearly depend on the dissociation kinetics through the Damköhler number, with different power laws in each wall that highlight the problem asymmetry.

In addition, in the case of chemo-attracted particles we find the additional requirement $Pe_{p}\gg {Da_{i}}^{1/2}$ for the model to be valid, since otherwise the particle boundary layer is not contained within the chemical boundary layer (i.e., δ_+_ ≪ *l*_+_ is not satisfied), and the underlying approximations of the model break down. In the case of chemo-repelled particles, however, we find that the scalings are such that this situation can never occur, so any value of *Pe*_p_ ≫ 1 is valid. More details about these conditions can be found in the Supporting Information.

### Water Recovery

Equipped with a quantitative theory to predict the accumulation of colloidal particles, we can now compute metrics relevant for colloid separation. We focus on the water recovery, typically defined in the literature^48^ as the proportion of freshwater that can be obtained from the contaminated feed which, as discussed above, has a (dimensionless) mean particle concentration of 1. Defining the freshwater stream as the region within the channel with particle concentration below a reference value *n*(*y*) < *n*_ref_, we can obtain the water recovery γ by plugging *n*(*y*_ref_) = *n*_ref_ into the expressions (eq 30a or 30b) for the particle distributions, with *y*_ref_ some position where the particle concentration reaches the reference concentration. Then, noting that γ = 1 – *y*_ref_ for chemo-attracted particles, while γ = *y*_ref_ if they are chemo-repelled, we can solve for γ. The result is

45a $$\gamma =1-\delta_{\pm }\log\left[\frac{1}{\delta_{\pm }n_{ref}}\right]\mathrm{for liquid sources}$$

and

45b $$\gamma =1-\delta_{\pm }\log^{1/2}\left[\frac{2}{\sqrt{\pi }\delta_{\pm }n_{ref}}\right]\mathrm{for gas sources}$$

## Microfluidic Experiments

### Materials and Methods

We fabricate microfluidic devices using standard soft lithography, where PDMS (Sylgard 184, Dow Corning) with a 10:1 ratio of elastomer base to curing agent is poured over a mold of SU-8 resin (SU-8 2050, Kayaku Advanced Materials) fabricated via photolithography. After curing, the solid polymer is peeled from the mold and bonded to a glass slide where a thin PDMS film has previously been spin-coated and cured. This setup ensures that all channel walls are made of PDMS, preventing any asymmetries in surface chemistry.

The chip, shown schematically in Figure 3a, consists of three parallel channels of nominal height and width of *H* = 40 μm and *W* = 250 μm, respectively. Flows of pure carbon dioxide and nitrogen gas are driven along the two side channels, while a suspension of deionized water (Direct Q3, Millipore) and fluorescent microparticles is injected into the central channel using a syringe pump (Pump 11 Pico Plus Elite, Harvard Apparatus). The channels are separated by walls of thickness *w* = 100 μm along a segment of length *L* = 55 mm, over which the natural permeability of the polymer to gases^49^ allows the CO_2_ to permeate from the “source” channel, dissolve and diffuse across the water, and then permeate again into the flowing nitrogen at the “sink”, replicating the setup outlined in Figure 1 with a monolithic, easy-to-fabricate PDMS chip.^22,24,26,35,36^ The sustained flow in all three channels ensures a persistent flux of dissolved CO_2_ in the spanwise direction, preventing the saturation of the sink with CO_2_ over time.

**Figure 3:**
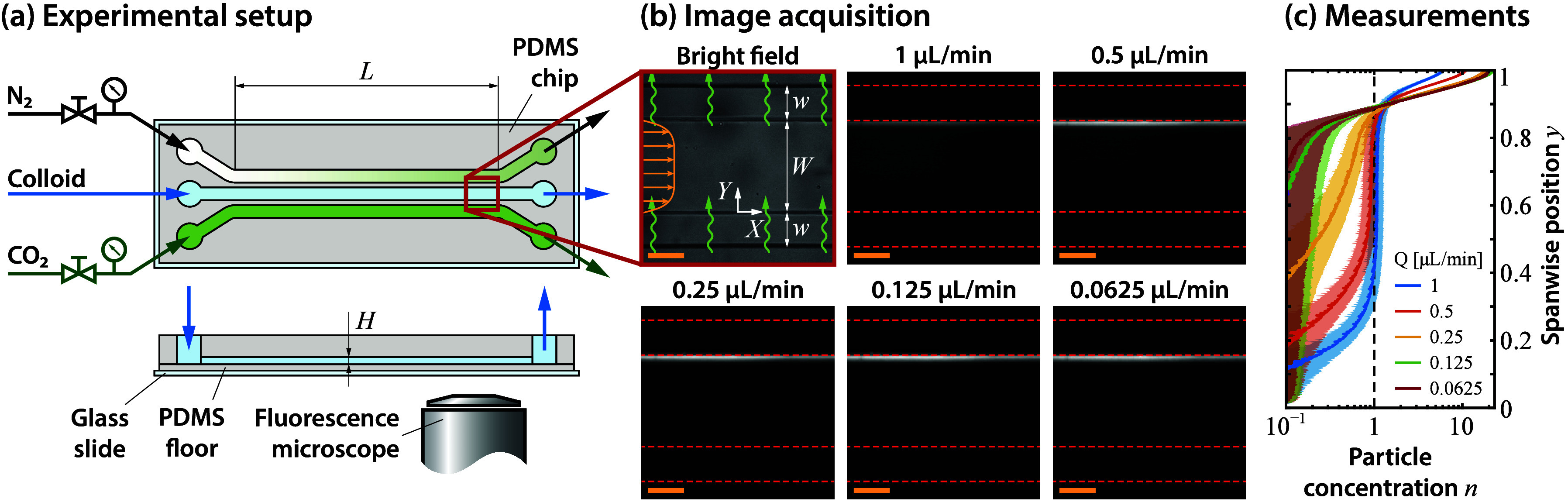
Microfluidic experiments reproduce the regime of fully developed particle concentrations using CO_2_-driven diffusiophoresis. (a) Sketches of the top view and cross-section of the microfluidic devices. (b) Bright-field image of the acquisition window, followed by the fluorescent signal acquired at steady state for five different flow rates. Note that the white particles (in this example, c-PS particles of diameter 0.1 μm) progressively accumulate at the top wall (i.e., the sink) with a decreasing flow rate. Scale bars on the bottom left corner correspond to 100 μm. (c) Corresponding fluorescent signal, normalized to yield a mean value of 1 (dashed line). Error bars indicate the standard deviation obtained from averaging the signal in time and in the streamwise direction. The data shows a thin particle-free boundary layer (i.e., an “exclusion zone”) near the source of CO_2_ that grows with a decreasing flow rate. The two curves with the lowest flow rates display a statistically negligible discrepancy, demonstrating that they correspond to the “fully-developed” regime of interest.

We note that even though our experiments are three-dimensional (with a channel height *H* that introduces vertical confinement) and the theory is formulated in two dimensions, they are still comparable. While vertical variations in the flow speed and subsequent Taylor dispersion induces vertical changes in the developing *c*_*s*_, *c*_*i*_, and *n* profiles, as shown by Shim et al.,^24^ in the fully developed case streamwise advection is zero and this effect is nonexistent. Furthermore, since the fluxes of solute, ions and particles through the top and bottom walls of the channels are either zero or can be assumed negligible compared to those through the sink and source side walls, we expect the *c*_*s*_, *c*_*i*_, and *n* profiles to be truly invariant in *x*, provided they have fully developed.

All experiments are performed setting a gauge pressure of *p*_in_ = 1.38 × 10^4^ Pa for both CO_2_ and N_2_ at the channel inlets, which leads to a concentration of *C*_*s*0_ ≈ 29.5 mM of CO_2_ dissolved in water at the “source” wall (see the Supporting Information). We then expect the CO_2_ dissolve in water through the dissociation reaction

46 $${\mathrm{CO}}_{2}+H_{2}O\overset{k_{f}}{\underset{k_{r}}{⇌}}H^{+}+{{\mathrm{HCO}}_{3}}^{-}$$

which sets the parameter ν = 2 from the reaction stoichiometry. Using well-characterized values of the reaction rate constants^50^ *k*_f_ and *k*_r_, we can estimate the scale for the ionic concentration *C*_*i*0_ ≈ 0.118 mM and the Damköhler numbers, as defined in eqs 21a and 21b, *Da*_*s*_ ≈ 1.28 and *Da*_*i*_ ≈ 306 in all experiments. These values correspond to the “weak dissociation” regime, as detailed in the Theory section above, and therefore do not replicate all the different theoretical regimes analyzed. Instead, the experiments focus on validating the specific case of a chemical source with a weakly dissociating gas. Since gas sources with a strong dissociation are predicted by our theory to yield a very low separation efficiency, we expect that the regime in the experiments is the most practically relevant to induce separation through diffusiophoresis using gases. In addition, under these conditions we can estimate the Debye length λ_D_ ≈ 29 nm, much smaller than any of the channel dimensions or particle sizes used.

We probe suspensions of three different types of particles: amine-modified polystyrene (a-PS), carboxylate-modified polystyrene (c-PS), and uncoated polystyrene (PS). Two different particles sizes are used for each of the three types, with their properties summarized in Table 2, allowing us to span a broad range of particle Péclet numbers in both chemo-attracted and chemo-repelled colloids. Since the particles size include small diameters below hundreds of nanometers, and since the ionic concentration is relatively low, we compute the mobilities Γ_p_ using the full expressions derived by Prieve et al.,^5^ which depend on both the particle size and the electrolyte concentration. The implications of choosing this model for the mobilities, which assumes a binary electrolyte and a constant potential on the particle, are discussed by Gupta et al.^37^ As opposed to the a-PS and PS particles, the c-PS particles increase their Γ_p_ in absolute value as the particle size decreases. The trend, opposite for all other particle types, can occur for highly negatively charged particles due to a subtle interplay between the electrophoretic and chemiphoretic contributions to the mobility.^26^ Particle diffusivities *D*_p_ are calculated from the Stokes–Einstein relation.^52^ In the experiments with amine-modified particles, we pump a 1 vol % aqueous solution of 3-aminopropyltriethoxysilane (APTES) for 15 min prior to injecting the article suspension to prevent particle adhesion with the PDMS walls.^25,26^ We use suspensions with a particle volume fraction of ϕ ≈ 1.5 × 10^–4^ in all experiments.

**Table 2:** Comparison of Different Particle Properties Used in the Experiments

particle type	amine-modified (a-PS)		carboxylate-modified (c-PS)		uncoated (PS)	
diameter (μm)	1	0.2	1	0.1	1.04	0.08
zeta potential ζ_p_ (mV)	60 (refs 14, 26, and 51 )		–75 (ref 26)		–50 (refs 14, 26, and 51 )	
mobility Γ_p_ (m^2^ s^–1^)	7.5 × 10^–10^	3.2 × 10^–10^	–6.1 × 10^–10^	–1.0 × 10^–9^	–4.6 × 10^–10^	–2.9 × 10^–10^
diffusivity *D*_p_ (m^2^ s^–1^)	4.4 × 10^–13^	2.2 × 10^–12^	4.4 × 10^–13^	4.4 × 10^–12^	4.2 × 10^–13^	5.4 × 10^–12^
Péclet number *Pe*_p_	1.7 × 10^3^	1.5 × 10^2^	1.4 × 10^3^	2.4 × 10^2^	1.1 × 10^3^	5.3 × 10^1^

### Experimental Protocol

We use an inverted fluorescence microscope (Leica DMI4000B) to image the central channel during a time interval of approximately 20 s, at either 10× or 20× magnification (window sizes of 1424 × 1064 μm and 712 × 532 μm, respectively). A typical acquisition image is shown in Figure 3b, for the case of 0.1 μm diameter c-PS particles. We use the fluorescence signal of the particles (displayed in white) as a proxy for their concentration, ensuring that the signal does not change appreciably during the acquisition time and is therefore in a steady state. For each type and size of particles, we also run a calibration test without CO_2_ or N_2_ to corroborate that the measured fluorescent signal changes linearly with concentration for the range of particle volume fractions that considered, as detailed in the Supporting Information.

As shown in Figure 3b, particles accumulate at the (top) sink wall, as expected for this particular case of chemo-repelled particles with a negative mobility Γ_p_. Since our goal is to capture the fully developed regime where particle profiles are invariant in the streamwise *X* direction, we fix the acquisition window at the end of the channel (*X* ≈ 55 mm), and progressively decrease the flow rate to reach the fully developed limit. Note that the flow rates used, shown in Figure 3b, are small (with *Q* = 0.0625–1 μL min^–1^) since experiments are constrained by the typical length of a glass slide (around 75 mm). In practically relevant scenarios, channel dimensions could be tuned to maximize flow rate while ensuring that the fully developed regime can be achieved within a feasible channel length, following the analysis in Shim et al. of the developing particle profiles.

Given that the width of the acquisition windows is much smaller than the channel length *L* ≈ 55 mm over which the particle concentration evolves in the streamwise direction, we average the fluorescence signal in *X* across the acquisition window, as well as in time. The result, normalized to ensure a unit mean concentration, is plotted in Figure 3c. For the highest three flow rates, the particle profiles are clearly still developing in the streamwise direction: they display a distinct particle free-exclusion zone near the (bottom) source wall, followed by a region of constant particle concentration in the intermediate section of the channel. This constant value is roughly the average *n* ≈ 1 incoming from the inlet, reinforcing our interpretation of a developing particle profile with a boundary-layer structure.^24^ In addition, these developing particle profiles display a peak at the (top) sink wall, which we attribute to the dissolved CO_2_ naturally present in the water diffusing into the absorbing sink, creating localized gradients that also induce diffusiophoresis. The particle profiles for the lowest two flow rates have no meaningful statistical difference, as evidenced in Figure 3c. This fact confirms that the profile has fully developed and that it can be compared against our theoretical model. The same process of varying the flow rate until a fully developed profile is identified is followed for all experiments, such that only the fully developed profiles are compared to our theory.

## Results and Discussion

The fully developed particle profiles obtained in six representative experiments are displayed in Figure 4a. Each of the six cases corresponds to particles of a different type or size, and is normalized to ensure a unit mean $\int_{0}^{1}ndy=1$ . In order to compare the profiles to the model, we focus on the maximum concentration *n*_max_ = *n*_±_(*y*_±_ = 0) at the walls, since a metric like the water recovery in eqs 45a and 45b, while highly relevant in practice, depends on an arbitrary reference particle concentration *n*_ref_ that is difficult to measure experimentally. From eq 30b and the expressions given by eqs 44a-b for δ_±_, we expect from theory that $n_{\max}=2/\left(\sqrt{\pi }\delta_{\pm }\right)$ and that

47a $$n_{\max}=\frac{2^{1/4}}{\pi^{1/2}}{{Da}_{i}}^{1/4}{{Pe}_{p}}^{1/2}\mathrm{at the source}$$

47b $$n_{\max}={\left(\frac{2\alpha }{\pi }\right)}^{1/2}{{Da}_{i}}^{2/5}{{Pe}_{p}}^{1/2}\mathrm{at the sink}$$

Qualitatively, we can expect that particles with a higher *Pe*_p_ will display higher *n*_max_, and that chemo-attracted particles will achieve lower concentrations than chemo-repelled particles at the same values of *Pe*_p_ since, comparing the prefactors, ${{Da}_{i}}^{2/5}>{{Da}_{i}}^{1/4}$ . This qualitative trend is reproduced in Figure 4a, where all particle types show a monotonic increase in *n*_max_ with *Pe*_p_, and where the chemo-repelled c-PS and PS particles show higher accumulation than the chemo-attracted a-PS particles.

**Figure 4:**
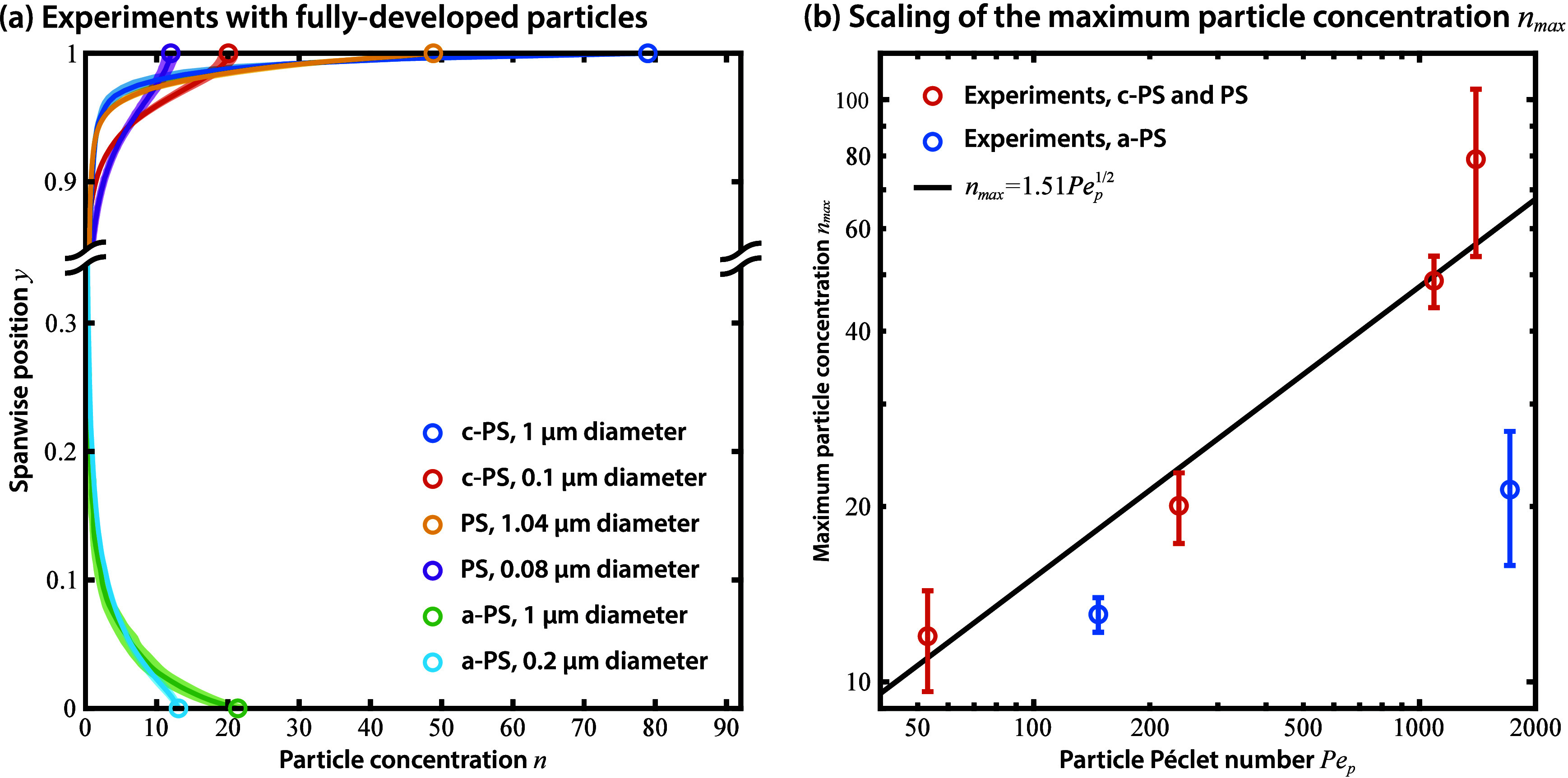
Experimental measurements of fully developed particle profiles reproduce the scaling predicted by theory. (a) Fluorescent signal, normalized to yield a mean value of 1, for fully developed profiles of particles of different types and sizes, as detailed in Table 2. (b) Maximum particle concentration *n*_max_ (at walls *y* = ±1) as a function of the particle Péclet number *Pe*_p_, as defined in eq 16. Note the trend $n_{\max}\propto {{Pe}_{p}}^{1/2}$ , predicted by theory, for chemo-repelled particles (c-PS and PS). For chemo-attracted particles (a-PS), the available experimental data displays significantly lower values of *n*_max_, agreeing qualitatively with theory.

The scaling $n_{\max}\propto {{Pe}_{p}}^{1/2}$ is also reproduced quantitatively for the four types of chemo-repelled particles, as shown in Figure 4b. While the availability of only two types of chemo-attracted particles precludes drawing any quantitative conclusion, the measured value of *n*_max_ is also consistently lower than the expected value for chemo-repelled particles of the same *Pe*_p_. It is worth noting that the prefactor of approximately 1.5 obtained from fitting the experimental data for chemo-repelled particles deviates from the expected value of $\sqrt{2\alpha /\pi }{{Da}_{i}}^{2/5}\approx 7.1$ by a factor of approximately 4.7. Such a deviation could originate from complexities in the charge dynamics at the particles, such as charge regulation mechanisms,^51^ from more complex reaction chemistry that is not captured by the single-reaction mechanism (eq 46), or due to the effect of particle–particle interactions that introduce dynamics beyond Brownian diffusion.^53^

The quantitative theory presented here is naturally subject to a number of limitations, the first being that it assumes an elementary dissociation reaction given by eq 1. While multi-ion electrolytes where concentrations of more than two species are non-negligible would introduce significant differences in the governing equations, complex reaction mechanisms, as long as they can be approximated by an effective single reaction of the form eq 1, would still be captured. Such effective reactions could arise, for instance, with the disparity of different reaction time scales that can be simplified using the well-known quasi-steady-state approximation (QSSA) in chemical kinetics. In fact, the dissociation of carbon dioxide given by eq 46 proceeds in two steps ${\mathrm{CO}}_{2}+H_{2}O⇌H_{2}{\mathrm{CO}}_{3}⇌H^{+}+{{\mathrm{HCO}}_{3}}^{-}$ , which can be approximated^54^ by the single reaction step given by eq 46.

Similarly, the model includes eq 5 for the diffusiophoretic velocity, which relies on a few assumptions. The first one is that the electrolyte can be regarded as binary, with one cationic and one anionic species being dominant over all other ions, which are assumed negligible in concentration. The expression implicitly relies on the fact that the electrolyte is fully and instantaneously dissociated in the thin electric double layer around the particles,^5^ which could be violated if the reaction terms (eq 1) become important enough to affect the Boltzmann distribution for the ions in that region. A scaling analysis of the governing equations for the ionic concentrations in the double layer, which we detail in the Supporting Information, reveals that eq 5 holds as long as

48 $$Da_{i}\ll {\left(\frac{W}{\lambda_{D}}\right)}^{2}$$

For the systems of interest, channel widths are not smaller than *W* = *O*(100 μm) and double layers are typically thinner than λ_D_ = *O*(0.1 μm), which leads to *Da*_*i*_ ≪ *O*(10^6^). We expect this bound to apply for most reactions of practical interest, although there could be extreme cases in which very large *Da*_*i*_ require a different theoretical description.

Our model also assumes a monodisperse distribution of particles, when true colloids (and especially environmental contaminants) are expected to be polydisperse. In the dilute limit ϕ ≪ 1, particle interactions can be neglected, and we expect that this model could still be easily applicable to a colloidal dispersion with a broad size distribution.

We also note that this theory is only concerned with the limit in which particle distribution is fully developed, i.e., it becomes invariant in the streamwise direction when sufficiently far downstream. Naturally, the downstream length scale over which the particle distribution reaches this fully developed state will also be key in designing separations driven by diffusiophoresis, since it would set a minimum design length to achieve the optimal water recovery given by eqs 45a-b. This length scale, obtained from balancing streamwise advection with cross-channel diffusiophoresis, can be estimated as *L*_dev_ ∼ *W* ^2^ *U*/Γ_p_, with *U* a typical velocity scale given by the streamwise flow rate. Further subtleties in the boundary layer analysis, such as the influence of spatially varying shear in rectangular channels, can be found in ref 24.

## Conclusion

We have developed a continuum theory to predict the maximum separation of a colloidal suspension driven by diffusiophoresis in a cross-channel electrolyte gradient. Three governing dimensionless parameters emerge from the theory: two Damköhler numbers *Da*_*s*_ and *Da*_*i*_, given by eqs 21a-b and comparing the rates of dissociation kinetics to the diffusion of the solute and resulting ions, and a particle Péclet number *Pe*_p_, given by eq 16 and comparing diffusiophoretic migration to the Brownian diffusion of the particles. The model predicts four distinct regimes (illustrated in Figure 2) depending on the mechanism by which the delivered chemical permeates into the channel (which we distinguish as either “liquid” or “gas” sources) and on whether its dissociation kinetics are “strong” (*Da*_*s*_ ≫ *Da*_*i*_) or “weak” (*Da*_*s*_ ≪ *Da*_*i*_) . All four regimes are governed by a single parameter δ measuring the width of the particle distribution, which displays distinct scalings in each regime as a function of *Pe*_p_ and, in certain regimes, also of *Da*_*i*_ and of a parameter ε that accounts for a small concentration at the chemical sink that may arise from imperfect transport through the channel walls. In addition, due to the asymmetry in the ion concentration and the boundary conditions at each wall, we distinguish between δ_+_ at the source (for chemo-attracted particles) and δ_–_ at the sink (for chemo-repelled particles), which in general have different scalings. From these expressions for δ_±_, we calculate the water recovery γ (eqs 45a-b) as a proxy of the colloid separation efficiency, although the insights about the structure of solutions that we have provided could be easily adapted to calculate other metrics quantifying separation. These metrics can then be used to design channel splittings that maximize the amount of filtrate (clean water) while minimizing the retentate (the particle-rich water stream in Figure 1b). All the expressions from the theory are summarized in Table 3, as well as the conditions of validity of the model. To conclude, we present microfluidic experiments using CO_2_-driven diffusiophoresis that confirm the theoretical scaling in one of these four regimes, using polystyrene particles with three different surface functionalization and with diameters ranging from 0.08 to 1 μm.

**Table 3:** Summary of Scaling Relations for the Nondimensional Width δ of the Particle Distributions, for All Four Identified Regimes, and Conditions of Validity of the Theory. All formulas assume *Da*_*s*_ = *O*(1)

	liquid source		gas source	
	chemo-attracted	chemo-repelled	chemo-attracted	chemo-repelled
strong dissociation	$\delta_{+}=\frac{{{Pe}_{p}}^{-1}}{1-\varepsilon^{1/\nu }}$	$\delta_{-}=\frac{\varepsilon^{1/\nu }{{Pe}_{p}}^{-1}}{1-\varepsilon^{1/\nu }}$	$\delta_{+}=2^{\left(2\nu -1\right)/2\nu }{{Da}_{i}}^{-1/2}{{Pe}_{p}}^{-1/2}$	$\delta_{-}=2^{\left(2\nu -1\right)/2\nu }{{Da}_{i}}^{-1/2}{{Pe}_{p}}^{-1/2}$
	*Pe*_p_ ≫ 1, *Da*_*i*_ ≪ 1	*Pe*_p_ ≫ 1, *Da*_*i*_ ≪ 1	$Pe_{p}\gg Da_{i}^{-1}\gg 1$	$Pe_{p}\gg Da_{i}^{-1}\gg 1$
weak dissociation	$\delta_{+}=\frac{\nu {{Pe}_{p}}^{-1}}{1-\varepsilon }$	$\delta_{-}=\frac{\nu \varepsilon {{Pe}_{p}}^{-1}}{1-\varepsilon }$	$\delta_{+}=2^{1/2}\nu^{1/4}{{Da}_{i}}^{-1/4}{{Pe}_{p}}^{-1/2}$	$\delta_{-}=2^{1/2}\alpha^{\left(1-\nu \right)/2}{{Da}_{i}}^{-\nu /\left(3\nu -1\right)}{{Pe}_{p}}^{-1/2}$
	*Pe*_p_ ≫ 1, *Da*_*i*_ ≫ 1	*Pe*_p_ ≫ 1, $Da_{i}\gg \varepsilon^{\left(1-3\nu \right)/\nu }\gg 1$	$$Pe_{p}\gg {{Da}_{i}}^{1/2}\gg 1$$	*Pe*_p_ ≫ 1, *Da*_*i*_ ≫ 1

Our analysis provides novel insights into the feasibility of diffusiophoresis as a method for colloid separation. First, it highlights that using a gas that strongly dissociates in water (*Da*_*i*_ ≪ *Da*_*s*_) leads to an unfavorable regime with low colloid separation, potentially unfeasible in applications. More importantly, it provides quantitative bounds for the separation efficiency in all regimes, essential to assess its potential scalability and design separations driven by diffusiophoresis.

## Supplementary Material



## Notes

Derivation of the leading-order particle profiles from the theory, detailed asymptotics for the ionic concentration profiles, and calibration of particle fluorescence intensity in the experiments (PDF)
