# Supplementary material for: Upper Bounds on the Colloid Separation Efficiency of Diffusiophoresis
Source: Langmuir. 2026 May 7;42(19):13306–18. doi: 10.1021/acs.langmuir.5c06768 (PMC13192318; doi:10.1021/acs.langmuir.5c06768)
Supplement: Supplementary file 1 [file la5c06768_si_001.pdf]

Supporting Information: Upper bounds on the colloid separation efficiency of diffusiophoresis

Fernando Temprano-Coleto,[†,‡] Jeongmin Kim,[†] Marcel M. Louis,[†] and Howard A. Stone[*,†]

† *Department of Mechanical and Aerospace Engineering, Princeton University, Princeton, NJ 08544, USA*

‡ *Andlinger Center for Energy and the Environment, Princeton University, Princeton, NJ 08544, USA*

E-mail: hastone@princeton.edu

DOCUMENT INFORMATION:

- Number of pages: 18
- Number of figures: 1
- Number of tables: 2

TABLE OF CONTENTS:

S1. Derivation of the leading-order particle profiles

S1.1. Liquid sources

S1.2. Gas sources

S2. Detailed asymptotics for the ionic concentration profiles

S2.1. Strong dissociation ($Da_i \ll Da_s$) - Gas sources

S2.2. Weak dissociation ($Da_i \gg Da_s$) - Liquid sources

S2.3. Weak dissociation ($Da_i \gg Da_s$) - Gas sources

S3. Effect of dissociation chemistry on electric double layers

S4. Outline of experimental parameters

S5. Calibration of particle fluorescence intensity

S1. Derivation of the leading-order particle profiles

S1.1. Liquid sources

We start by combining the exact solution for the particle concentrations [Equation (19) in the main text] with the Taylor expansions for c_i for liquid sources [Equation (28) in the main text]. After factoring out $c_i(0)$ from the numerator and denominator, and noting that the derivative at walls is negative at the source ($\mathrm{d}c_i/\mathrm{d}y_+ < 0$ at $y_+ = 0$) and positive at the sink ($\mathrm{d}c_i/\mathrm{d}y_- > 0$ at $y_- = 0$), we obtain

$$n_\pm = \frac{\left[1 \mp \dfrac{1}{c_i(0)}\left|\dfrac{\mathrm{d}c_i}{\mathrm{d}y_\pm}(0)\right| y_\pm + O(y_\pm^2)\right]^{\pm Pe_p}}{\displaystyle\int_0^1 \left[1 \mp \dfrac{1}{c_i(0)}\left|\dfrac{\mathrm{d}c_i}{\mathrm{d}y_\pm}(0)\right| y_\pm + O(y_\pm^2)\right]^{\pm Pe_p} \mathrm{d}y_\pm}. \tag{S1}$$

We then seek to find approximations for this expression at distances sufficiently close to the walls, where $y_\pm$ is sufficiently small. To that end, we define $y_\pm := \delta_\pm \xi_\pm$ as a rescaled wall coordinate, such that $\xi_\pm = O(1)$ wherever $y_\pm = O(\delta_\pm)$, and with $\delta_\pm$ the (small) boundary layer thickness. This leads to

$$n_\pm = \frac{\left[1 \mp \dfrac{1}{c_i(0)}\left|\dfrac{\mathrm{d}c_i}{\mathrm{d}y_\pm}(0)\right| \delta_\pm \xi_\pm + O(\xi_\pm^2)\right]^{\pm Pe_p}}{\displaystyle\int_0^1 \left[1 \mp \dfrac{1}{c_i(0)}\left|\dfrac{\mathrm{d}c_i}{\mathrm{d}y_\pm}(0)\right| \delta_\pm \xi_\pm + O(\xi_\pm^2)\right]^{\pm Pe_p} \mathrm{d}y_\pm}. \tag{S2}$$

Since we expect a large absolute value of the exponent $Pe_p \gg 1$, we expect the terms of the expansion to be small if the boundary layer thickness is

$$\delta_\pm = \frac{c_i(0)}{\left|\dfrac{\mathrm{d}c_i}{\mathrm{d}y_\pm}(0)\right|} Pe_p^{-1}, \tag{S3}$$

such that

$$n_\pm = \frac{\left[1 \mp \dfrac{\xi_\pm}{Pe_p} + O(Pe_p^{-2})\right]^{\pm Pe_p}}{\delta_\pm \displaystyle\int_0^{1/\delta_\pm} \left[1 \mp \dfrac{\xi_\pm}{Pe_p} + O(Pe_p^{-2})\right]^{\pm Pe_p} \mathrm{d}\xi_\pm}. \tag{S4}$$

Since the Péclet number is large, we can then invoke that $\left[1 \mp \frac{\xi_\pm}{Pe_p} + O(Pe_p^{-2})\right]^{\pm Pe_p} = e^{-\xi_\pm}\left[1 + O\big(Pe_p^{-1}\big)\right]$ and, after integrating, obtain

$$n_\pm = \frac{e^{-\xi_\pm}}{\delta_\pm\left(1 - e^{-1/\delta_\pm}\right)} + o\big(\delta_\pm^{-1}\big) \ , \tag{S5}$$

which, after neglecting exponentially small terms and changing variables back to $y_\pm$, results in

$$n_\pm = \frac{e^{-y_\pm/\delta_\pm}}{\delta_\pm} + o\big(\delta_\pm^{-1}\big) \ , \tag{S6}$$

whose leading-order term $n_\pm^{(0)}$ is given by Equation (30a) in the main text.

S1.2. Gas sources

We can follow an analogous procedure combining the exact solution for the particles [Equation (19) in the main text] with the Taylor expansions for c_i for gas sources [Equation (29) in the main text]. After factoring out $c_i(0)$ form the numerator and denominator, and noting that the second derivative at walls is negative at the source ($\mathrm{d}^2c_i/\mathrm{d}y_+^2 < 0$ at $y_+ = 0$) and positive at the sink ($\mathrm{d}^2c_i/\mathrm{d}y_-^2 > 0$ at $y_- = 0$), we obtain

$$n_\pm = \frac{\left[1 \mp \frac{1}{c_i(0)}\left|\frac{\mathrm{d}^2c_i}{\mathrm{d}y_\pm^2}(0)\right|\frac{y_\pm^2}{2} + O(y_\pm^3)\right]^{\pm Pe_p}}{\int_0^1 \left[1 \mp \frac{1}{c_i(0)}\left|\frac{\mathrm{d}^2c_i}{\mathrm{d}y_\pm^2}(0)\right|\frac{y_\pm^2}{2} + O(y_\pm^3)\right]^{\pm Pe_p} \mathrm{d}y_\pm} . \tag{S7}$$

Defining $y_\pm := \delta_\pm\xi_\pm$ with a boundary layer thickness $\delta_\pm$ such that

$$\delta_\pm = \left[\frac{2c_i(0)}{\left|\frac{\mathrm{d}^2c_i}{\mathrm{d}y_\pm^2}(0)\right|}\right]^{1/2} Pe_p^{-1/2}, \tag{S8}$$

we obtain

$$n_\pm = \frac{\left[1 \mp \frac{\xi_\pm^2}{Pe_p} + O(Pe_p^{-2})\right]^{\pm Pe_p}}{\delta_\pm \int_0^{1/\delta_\pm} \left[1 \mp \frac{\xi_\pm^2}{Pe_p} + O(Pe_p^{-2})\right]^{\pm Pe_p} \mathrm{d}\xi_\pm} . \tag{S9}$$

After noting $\left[1 \mp \frac{\xi_\pm^2}{Pe_p} + O(Pe_p^{-2})\right]^{\pm Pe_p} = e^{-\xi_\pm^2}\left[1 + O\left(Pe_p^{-1}\right)\right]$, integrating, and neglecting exponentially small terms, we obtain

$$n_\pm = \frac{2e^{-\left(\frac{y_\pm}{\delta_\pm}\right)^2}}{\delta_\pm\sqrt{\pi}} + o\left(\delta_\pm^{-1}\right) \ , \tag{S10}$$

whose leading-order term $n_\pm^{(0)}$ is given by Equation (30b) of the main text.

S2. Detailed asymptotics for the ionic concentration profiles

S2.1. Strong dissociation ($Da_i \ll Da_s$) - Gas sources

Assuming $Da_s = O(1)$ and $Da_i \ll 1$, we insert the expansion $c_i = c_i^{(0)} + Da_i c_i^{(1)} + O\left(Da_i^2\right)$ into Equation (23) of the main text, we obtain, at leading order,

$$\frac{\mathrm{d}^4 c_i^{(0)}}{\mathrm{d}y^4} = Da_s \frac{\mathrm{d}^2 c_i^{(0)}}{\mathrm{d}y^2}, \tag{S11}$$

which has a solution

$$c_i^{(0)} = A + B\,y + Ce^{-\sqrt{Da_s}y} + De^{\sqrt{Da_s}y}, \tag{S12}$$

and with A, B, C, D constants. The boundary conditions given by (25) in the main text result in

$$\frac{\mathrm{d}^2 c_i^{(0)}}{\mathrm{d}y^2}(0) = \frac{\mathrm{d}^2 c_i^{(0)}}{\mathrm{d}y^2}(1) = \frac{\mathrm{d}c_i^{(0)}}{\mathrm{d}y}(0) = \frac{\mathrm{d}c_i^{(0)}}{\mathrm{d}y}(1) = 0 \tag{S13}$$

at leading order, which give $B = C = D = 0$ and $c_i^{(0)} = A$, with A undetermined.

At first order in Da_i, the governing equation is the same as in the zeroth-order case, i.e.

$$\frac{\mathrm{d}^4 c_i^{(1)}}{\mathrm{d}y^4} = Da_s \frac{\mathrm{d}^2 c_i^{(1)}}{\mathrm{d}y^2}, \tag{S14}$$

with boundary conditions

$$\frac{\mathrm{d}^2 c_i^{(1)}}{\mathrm{d}y^2} = \left(c_i^{(0)}\right)^{\nu} - 1 = A^{\nu} - 1 \quad \text{at } y = 0, \tag{S15a}$$

$$\frac{\mathrm{d}^2 c_i^{(1)}}{\mathrm{d}y^2} = \left(c_i^{(0)}\right)^{\nu} = A^{\nu} \quad \text{at } y = 1, \tag{S15b}$$

$$\frac{\mathrm{d}c_i^{(1)}}{\mathrm{d}y} = 0 \quad \text{at } y = 0, \tag{S15c}$$

$$\frac{\mathrm{d}c_i^{(1)}}{\mathrm{d}y} = 0 \quad \text{at } y = 1. \tag{S15d}$$

The general solution of (S14) is $c_i^{(1)} = E + F\,y + Ge^{-\sqrt{Da_s}y} + He^{\sqrt{Da_s}y}$ and, after applying the boundary conditions (S15), we obtain

$$A = 2^{-1/\nu}, \tag{S16a}$$

$$F = -\frac{\coth\left(\sqrt{Da_s}/2\right)}{2\sqrt{Da_s}}, \tag{S16b}$$

$$G = -\frac{e^{\sqrt{Da_s}/2}}{4\,Da_s \sinh\left(\sqrt{Da_s}/2\right)}, \tag{S16c}$$

$$H = \frac{e^{-\sqrt{Da_s}/2}}{4\,Da_s \sinh\left(\sqrt{Da_s}/2\right)}, \tag{S16d}$$

with E remaining a free parameter at this order. To obtain it, we solve the second order problem given by

$$\frac{\mathrm{d}^4 c_i^{(2)}}{\mathrm{d}y^4} - Da_s \frac{\mathrm{d}^2 c_i^{(2)}}{\mathrm{d}y^2} = \nu\, 2^{\frac{(1-\nu)}{\nu}} \frac{\mathrm{d}^2 c_i^{(1)}}{\mathrm{d}y^2}, \tag{S17}$$

with boundary conditions

$$\frac{\mathrm{d}^2 c_i^{(2)}}{\mathrm{d}y^2} = \nu\, 2^{\frac{(1-\nu)}{\nu}} c_i^{(1)} \quad \text{at } y = 0, \tag{S18a}$$

$$\frac{\mathrm{d}^2 c_i^{(2)}}{\mathrm{d}y^2} = \nu\, 2^{\frac{(1-\nu)}{\nu}} c_i^{(1)} \quad \text{at } y = 1, \tag{S18b}$$

$$\frac{\mathrm{d}c_i^{(2)}}{\mathrm{d}y} = 0 \quad \text{at } y = 0, \tag{S18c}$$

$$\frac{\mathrm{d}c_i^{(2)}}{\mathrm{d}y} = 0 \quad \text{at } y = 1, \tag{S18d}$$

which leads to $E = \coth\left(\sqrt{Da_s}/2\right) / \left(4\sqrt{Da_s}\right)$. All these constants lead to the solutions $c_i^{(0)}$ and $c_i^{(1)}$ given by Equations (36) in the main text.

S2.2. Weak dissociation ($Da_i \gg Da_s$) - Liquid sources

Assuming $Da_s = O(1)$ and $Da_i \gg 1$, we insert the expansion $c_i = c_i^{(0)} + Da_i^{-1} c_i^{(1)} + O(Da_i^{-2})$ into Equation (23) of the main text, we obtain

$$\frac{\mathrm{d}^2}{\mathrm{d}y^2}\left[\left(c_i^{(0)}\right)^{\nu}\right] = 0, \tag{S19a}$$

$$\frac{\mathrm{d}^2}{\mathrm{d}y^2}\left[\nu\left(c_i^{(0)}\right)^{\nu-1} c_i^{(1)}\right] = \frac{\mathrm{d}^4 c_i^{(0)}}{\mathrm{d}y^4} - Da_s \frac{\mathrm{d}^2 c_i^{(0)}}{\mathrm{d}y^2}, \tag{S19b}$$

at zeroth and first order, respectively, which have solutions given by

$$c_i^{(0)} = [A + By]^{1/\nu}, \tag{S20a}$$

$$c_i^{(1)} = -\frac{B^2(\nu-1)}{\nu^3}[A+By]^{\frac{2-3\nu}{\nu}} - \frac{Da_s}{\nu}[A+By]^{\frac{2-\nu}{\nu}} + \frac{C+Dy}{\nu}[A+By]^{\frac{1-\nu}{\nu}}, \tag{S20b}$$

However, these are only an "outer" solutions since they cannot satisfy all four boundary conditions given by (24) at each order simultaneously.

To find the solution close to the domain boundaries, we first introduce a rescaled "inner" coordinate y_B near the (bottom) $y = 0$ wall, such that

$$y_B = Da_i^m y, \tag{S21}$$

and introduce it into the governing equation (23) to obtain the inner equation

$$\frac{\mathrm{d}^4 c_{iB}}{\mathrm{d}y_B^4} = Da_i^{1-2m}\frac{\mathrm{d}^2}{\mathrm{d}y_B^2}\left[(c_{iB})^{\nu}\right] + Da_s Da_i^{-2m}\frac{\mathrm{d}^2 c_{iB}}{\mathrm{d}y_B^2}, \tag{S22}$$

where we have denoted the inner solution as c_{iB} for notational convenience. The value $m = 1/2$ is required to retain a meaningful dominant balance of terms, which leads to

$$\frac{\mathrm{d}^4 c_{iB}}{\mathrm{d}y_B^4} = \frac{\mathrm{d}^2}{\mathrm{d}y_B^2}\left[(c_{iB})^{\nu}\right] + Da_s Da_i^{-1}\frac{\mathrm{d}^2 c_{iB}}{\mathrm{d}y_B^2}, \tag{S23}$$

which must be solved order by order imposing the boundary conditions at $y_B = 0$ and matching to the outer solution as $y_B \to \infty$. To find out the terms in the matching, we expand the outer solution in the inner

coordinate y_B as

$$\begin{aligned} c_i(y_B) &= c_i^{(0)}(y_B) + Da_i^{-1} c_i^{(1)}(y_B) + O(Da_i^{-2}) = \\ &= [A + Da_i^{-1/2} B y_B]^{1/\nu} + Da_i^{-1} c_i^{(1)}(y_B) + O(Da_i^{-2}) = \\ &= A^{1/\nu} + Da_i^{-1/2} \frac{A^{\frac{1-\nu}{\nu}} B}{\nu} y_B + Da_i^{-1} \left[-\frac{A^{\frac{1-2\nu}{\nu}} B^2 (\nu - 1)}{2\nu^2} y_B^2 + c_i^{(1)}(y_B = 0) \right] + O\left(Da_i^{-3/2}\right). \end{aligned} \tag{S24}$$

We then pose an expansion of the inner solution $c_{iB} = c_{iB}^{(0)} + Da_i^{-1/2} c_{iB}^{(1)} + Da_i^{-1} c_{iB}^{(2)} + O\left(Da_i^{-3/2}\right)$ and insert it in the inner equation (S23) and in the boundary conditions to obtain the inner problem at each order.

At zeroth order, we have

$$\frac{\mathrm{d}^4 c_{iB}^{(0)}}{\mathrm{d}y_B^4} = \frac{\mathrm{d}^2}{\mathrm{d}y_B^2} \left[\left(c_{iB}^{(0)} \right)^{\nu} \right], \tag{S25a}$$

$$c_{iB}^{(0)} = 1 \text{ at } y_B = 0, \tag{S25b}$$

$$\frac{\mathrm{d}^2 c_{iB}^{(0)}}{\mathrm{d}y_B^2} = 0 \text{ at } y_B = 0, \tag{S25c}$$

$$c_{iB}^{(0)} \to A^{1/\nu} \text{ as } y_B \to \infty, \tag{S25d}$$

which requires the constant to be $A = 1$ and results in $c_{iB}^{(0)} = 1$.

At order $Da_i^{-1/2}$, we find

$$\frac{\mathrm{d}^4 c_{iB}^{(1)}}{\mathrm{d}y_B^4} = \nu \frac{\mathrm{d}^2 c_{iB}^{(1)}}{\mathrm{d}y_B^2}, \tag{S26a}$$

$$c_{iB}^{(1)} = 0 \text{ at } y_B = 0, \tag{S26b}$$

$$\frac{\mathrm{d}^2 c_{iB}^{(1)}}{\mathrm{d}y_B^2} = 0 \text{ at } y_B = 0, \tag{S26c}$$

$$c_{iB}^{(1)} \to \frac{B}{\nu} y_B \text{ as } y_B \to \infty, \tag{S26d}$$

which has solution $c_{iB}^{(1)} = \dfrac{B}{\nu} y_B$.

At order Da_i^{-1}, we find

$$\frac{\mathrm{d}^4 c_{iB}^{(2)}}{\mathrm{d}y_B^4} = \nu \frac{\mathrm{d}^2}{\mathrm{d}y_B^2}\left[\frac{(\nu-1)B^2}{2\nu^2} y_B^2 + c_{iB}^{(2)}\right], \tag{S27a}$$

$$c_{iB}^{(2)} = 0 \text{ at } y_B = 0, \tag{S27b}$$

$$\frac{\mathrm{d}^2 c_{iB}^{(2)}}{\mathrm{d}y_B^2} = 0 \text{ at } y_B = 0, \tag{S27c}$$

$$c_{iB}^{(2)} \to -\frac{(\nu-1)B^2}{\nu^3}\left[\frac{\nu}{2}y_B^2 + 1\right] + \frac{C - Da_s}{\nu} \text{ as } y_B \to \infty, \tag{S27d}$$

which has solution $c_{iB}^{(2)} = \frac{(\nu-1)B^2}{\nu^3}\left[e^{-\sqrt{\nu}y_B} - \frac{\nu}{2}y_B^2 - 1\right]$ and fixes $C = Da_s$.

We can then proceed analogously with the boundary-layer analysis of the (top) $y = 1$ wall. Introducing a rescaled "inner" coordinate y_T such that

$$y_T = Da_i^{1/2}(1 - y), \tag{S28}$$

and plugging it into Equation (23) of the main text, we obtain the inner equation

$$\frac{\mathrm{d}^4 c_{iT}}{\mathrm{d}y_T^4} = \frac{\mathrm{d}^2}{\mathrm{d}y_T^2}\left[(c_{iT})^\nu\right] + Da_s Da_i^{-1}\frac{\mathrm{d}^2 c_{iT}}{\mathrm{d}y_T^2}. \tag{S29}$$

Expanding the outer solution in the inner coordinate y_T, we arrive at

$$\begin{aligned}
c_i(y_T) &= c_i^{(0)}(y_T) + Da_i^{-1}c_i^{(1)}(y_T) + O(Da_i^{-2}) = \\
&= \left[(1+B) - Da_i^{-1/2}By_T\right]^{1/\nu} + Da_i^{-1}c_i^{(1)}(y_T) + O(Da_i^{-2}) = \\
&= (1+B)^{1/\nu} - Da_i^{-1/2}\frac{B(1+B)^{\frac{1-\nu}{\nu}}}{\nu}y_T + \\
&+ Da_i^{-1}\left[-\frac{(\nu-1)B^2(1+B)^{\frac{1-2\nu}{\nu}}}{2\nu^2}y_T^2 + c_i^{(1)}(y_T = 0)\right] + O\left(Da_i^{-3/2}\right),
\end{aligned} \tag{S30}$$

where we have already used $A = 1$ obtained from the matching conditions at the bottom $y = 0$ boundary.

Then, we insert an expansion of the inner solution $c_{iT} = c_{iT}^{(0)} + Da_i^{-1/2}c_{iT}^{(1)} + Da_i^{-1}c_{iT}^{(2)} + O\left(Da_i^{-3/2}\right)$ in the inner equation (S29) and in the boundary conditions to obtain the inner problem at each order.

At zeroth order, we have

$$\frac{\mathrm{d}^4 c_{iT}^{(0)}}{\mathrm{d}y_T^4} = \frac{\mathrm{d}^2}{\mathrm{d}y_T^2}\left[\left(c_{iT}^{(0)}\right)^{\nu}\right], \tag{S31a}$$

$$c_{iT}^{(0)} = \varepsilon^{1/\nu} \text{ at } y_T = 0, \tag{S31b}$$

$$\frac{\mathrm{d}^2 c_{iT}^{(0)}}{\mathrm{d}y_T^2} = 0 \text{ at } y_T = 0, \tag{S31c}$$

$$c_{iT}^{(0)} \to (1+B)^{1/\nu} \text{ as } y_T \to \infty, \tag{S31d}$$

which requires the constant to be $B = \varepsilon - 1$ and results in $c_{iT}^{(0)} = \varepsilon^{1/\nu}$.

At order $Da_i^{-1/2}$, we find

$$\frac{\mathrm{d}^4 c_{iT}^{(1)}}{\mathrm{d}y_T^4} = \nu\varepsilon^{\frac{\nu-1}{\nu}} \frac{\mathrm{d}^2 c_{iT}^{(1)}}{\mathrm{d}y_T^2}, \tag{S32a}$$

$$c_{iT}^{(1)} = 0 \text{ at } y_T = 0, \tag{S32b}$$

$$\frac{\mathrm{d}^2 c_{iT}^{(1)}}{\mathrm{d}y_T^2} = 0 \text{ at } y_T = 0, \tag{S32c}$$

$$c_{iT}^{(1)} \to \frac{(1-\varepsilon)\,\varepsilon^{\frac{1-\nu}{\nu}}}{\nu} y_T \text{ as } y_T \to \infty, \tag{S32d}$$

with solution $c_{iT}^{(1)} = \dfrac{(1-\varepsilon)\,\varepsilon^{\frac{1-\nu}{\nu}}}{\nu} y_T$.

At order Da_i^{-1}, we find

$$\frac{\mathrm{d}^4 c_{iT}^{(1)}}{\mathrm{d}y_T^4} = \nu\varepsilon^{\frac{\nu-1}{\nu}} \frac{\mathrm{d}^2}{\mathrm{d}y_T^2}\left[\frac{(\nu-1)(1-\varepsilon)^2\,\varepsilon^{\frac{1-2\nu}{\nu}}}{2\nu^2} y_T^2 + c_{iT}^{(2)}\right], \tag{S33a}$$

$$c_{iT}^{(2)} = 0 \text{ at } y_T = 0, \tag{S33b}$$

$$\frac{\mathrm{d}^2 c_{iT}^{(2)}}{\mathrm{d}y_T^2} = 0 \text{ at } y_T = 0, \tag{S33c}$$

$$c_{iT}^{(2)} \to -\frac{(\nu-1)(1-\varepsilon)^2\,\varepsilon^{\frac{1-2\nu}{\nu}}}{\nu^3}\left[\frac{\nu}{2} y_T^2 + \varepsilon^{1/\nu}\right] + \frac{\varepsilon^{\frac{1-\nu}{\nu}}}{\nu}\left[D + Da_s(1-\varepsilon)\right] \text{ as } y_T \to \infty, \tag{S33d}$$

which has solution $c_{iT}^{(2)} = \dfrac{(\nu-1)(1-\varepsilon)^2 \varepsilon^{\frac{2-3\nu}{\nu}}}{\nu^3}\left[e^{-\sqrt{\nu}\varepsilon^{\frac{\nu-1}{2\nu}} y_T} - \dfrac{\nu\varepsilon^{\frac{\nu-1}{\nu}}}{2} y_T^2 - 1\right]$ and fixes $D = -Da_s(1-\varepsilon)$.

In summary, the solutions near the bottom and top walls can then be expressed as

$$c_{iB} = 1 - Da_i^{-1/2}\frac{(1-\varepsilon)}{\nu}y_B + Da_i^{-1}\frac{(\nu-1)(1-\varepsilon)^2}{\nu^3}\left[e^{-\sqrt{\nu}y_B} - \frac{\nu}{2}y_B^2 - 1\right] + O\left(Da_i^{-3/2}\right), \tag{S34a}$$

$$\begin{aligned} c_{iT} = {} & \varepsilon^{1/\nu} - Da_i^{-1/2}\frac{(1-\varepsilon)\varepsilon^{\frac{1-\nu}{\nu}}}{\nu}y_T + \\ & + Da_i^{-1}\frac{(\nu-1)(1-\varepsilon)^2\varepsilon^{\frac{2-3\nu}{\nu}}}{\nu^3}\left[e^{-\sqrt{\nu}\varepsilon^{\frac{\nu-1}{2\nu}}y_T} - \frac{\nu\varepsilon^{\frac{\nu-1}{\nu}}}{2}y_T^2 - 1\right] + O\left(Da_i^{-3/2}\right), \end{aligned} \tag{S34b}$$

which can be re-expressed in terms of the outer variables $y_+ = Da_i^{1/2}y_B$ and $y_- = Da_i^{1/2}y_T$ and then expanded up to linear order to obtain

$$c_i \approx 1 - \frac{(1-\varepsilon)}{\nu}\left[1 + Da_i^{-1/2}\frac{(\nu-1)(1-\varepsilon)}{\nu^{3/2}}\right]y_+ \qquad \text{at the source,} \tag{S35a}$$

$$c_i \approx \varepsilon^{1/\nu} + \frac{(1-\varepsilon)\varepsilon^{\frac{1-\nu}{\nu}}}{\nu}\left[1 - Da_i^{-1/2}\frac{(\nu-1)(1-\varepsilon)\varepsilon^{\frac{1-3\nu}{2\nu}}}{\nu^{3/2}}\right]y_- \qquad \text{at the sink.} \tag{S35b}$$

After neglecting terms of order $Da_i^{-1/2}$, the above expressions (S35) simplify into Equations (39) in the main text. It is worth remarking that, since $\nu > 1$ and $0 < \varepsilon < 1$, the $O\left(Da_i^{-1/2}\right)$ term in (S35a) can always be neglected for sufficiently large $Da_i \gg 1$. However, the expression $\varepsilon^{\frac{1-3\nu}{2\nu}}$ could become large if $\varepsilon \ll 1$, potentially compensating the small $Da_i^{-1/2}$ and invalidating the asymptotic expansion. This breakdown of the theory stems from the implicit assumption that $\varepsilon = O(1)$. Since a more careful analysis for a small $\varepsilon \ll 1$ would be out of the scope of this paper, we simply remark that the theory will be valid as long as

$$Da_i \gg \frac{(\nu-1)^2}{\nu^3}(1-\varepsilon)^2\varepsilon^{\frac{1-3\nu}{\nu}}, \tag{S36}$$

under which the $O(Da_i^{-1/2})$ term in (S35b) can clearly be safely neglected.

S2.3. Weak dissociation ($Da_i \gg Da_s$) - Gas sources

In the case of gas sources, the only difference with respect to the liquid sources is the boundary conditions. Assuming $Da_s = O(1)$ and $Da_i \gg 1$, we pose an expansion $c_i = c_i^{(0)} + Da_i^{-1}c_i^{(1)} + O(Da_i^{-2})$, insert it in Equation (23) of the main text, and obtain the same outer solutions

$$c_i^{(0)} = [A + By]^{1/\nu}, \tag{S37a}$$

$$c_i^{(1)} = -\frac{B^2(\nu-1)}{\nu^3}[A+By]^{\frac{2-3\nu}{\nu}} - \frac{Da_s}{\nu}[A+By]^{\frac{2-\nu}{\nu}} + \frac{C+Dy}{\nu}[A+By]^{\frac{1-\nu}{\nu}}, \tag{S37b}$$

at zeroth and first order, respectively. Note that these are identical to the outer solution for the case of a liquid source (S20).

To find the inner solution close to the bottom $y = 0$ boundary, we introduce the same rescaled "inner" coordinate y_B near the (bottom) $y = 0$ wall as in the case for liquid sources, such that

$$y_B = Da_i^{1/2} y, \tag{S38}$$

which, once introduced in the governing equation (23), leads to a hierarchy of inner problems analogous to those of the case with liquid sources (S25) and (S26), with the same matching conditions but with different boundary conditions at the bottom wall $y_B = 0$.

At zeroth order, the inner problem is

$$\frac{\mathrm{d}^4 c_{iB}^{(0)}}{\mathrm{d}y_B^4} = \frac{\mathrm{d}^2}{\mathrm{d}y_B^2}\left[\left(c_{iB}^{(0)}\right)^{\nu}\right], \tag{S39a}$$

$$\left(c_{iB}^{(0)}\right)^{\nu} - \frac{\mathrm{d}^2 c_{iB}^{(0)}}{\mathrm{d}y_B^2} = 1 \text{ at } y_B = 0, \tag{S39b}$$

$$\frac{\mathrm{d}c_{iB}^{(0)}}{\mathrm{d}y_B} = 0 \text{ at } y_B = 0, \tag{S39c}$$

$$c_{iB}^{(0)} \to A^{1/\nu} \text{ as } y_B \to \infty, \tag{S39d}$$

which leads to $A = 1$ and to a solution $c_{iB}^{(0)} = 1$.

At order $Da_i^{-1/2}$, the inner problem is

$$\frac{\mathrm{d}^4 c_{iB}^{(1)}}{\mathrm{d}y_B^4} = \nu \frac{\mathrm{d}^2 c_{iB}^{(1)}}{\mathrm{d}y_B^2}, \tag{S40a}$$

$$\nu c_{iB}^{(1)} - \frac{\mathrm{d}^2 c_{iB}^{(1)}}{\mathrm{d}y_B^2} = 0 \text{ at } y_B = 0, \tag{S40b}$$

$$\frac{\mathrm{d}^1 c_{iB}^{(1)}}{\mathrm{d}y_B^1} = 0 \text{ at } y_B = 0, \tag{S40c}$$

$$c_{iB}^{(1)} \to \frac{B}{\nu} y_B \text{ as } y_B \to \infty, \tag{S40d}$$

which has solution $c_{iB}^{(1)} = \dfrac{B}{\nu}\left[y_B + \dfrac{e^{-\sqrt{\nu} y_B}}{\nu^{1/2}}\right]$.

To find the inner solution close to the top $y = 1$ boundary, we need a different boundary layer rescaling, since a naive $y_T = Da_i^{1/2}(1 - y)$ fails to produce a consistent leading-order inner problem. The reason for this is that, unlike the case of a liquid source where $c_i(y = 1) \to \varepsilon$ as $Da_i \to \infty$, for a gas source we have that $c_i(y = 1) \to 0$ as $Da_i \to \infty$, and therefore $c_i(y = 1)$ can get arbitrarily small and must also be rescaled

by a small parameter to arrive at a consistent inner problem. We therefore pose a scaling

$$y_T = Da_i^m(1-y), \tag{S41a}$$

$$c_{iT} = Da_i^n c_i, \tag{S41b}$$

which, introduced in Equation (23) of the main text, leads to

$$\frac{\mathrm{d}^4 c_{iT}}{\mathrm{d}y_T^4} = Da_i^{1-2m-(\nu-1)n}\frac{\mathrm{d}^2}{\mathrm{d}y_T^2}\left[(c_{iT})^\nu\right] + Da_s Da_i^{-2m}\frac{\mathrm{d}^2 c_{iT}}{\mathrm{d}y_T^2}. \tag{S42}$$

Similarly, this rescaling turns the boundary conditions (25b) and (25c) of the main text into

$$Da_i^{1-2m-(\nu-1)n} c_{iT}^\nu - \frac{\mathrm{d}^2 c_{iT}}{\mathrm{d}y_T^2} = 0, \tag{S43a}$$

$$\frac{\mathrm{d}c_{iT}}{\mathrm{d}y_T} = 0. \tag{S43b}$$

We therefore set

$$n = \frac{1-2m}{\nu-1} \tag{S44}$$

to achieve a meaningful dominant balance. We can then determine the value of m by examining the matching condition: expressing the outer solution in terms of the inner variable y_T and noting that $A = 1$ from the previous analysis, we obtain

$$c_i(y_T) = \left[(1+B) - Da_i^{-m} B y_T\right]^{1nu} + O(Da_i^{-1}). \tag{S45}$$

The matching condition dictates that, if $B \neq 0$, the inner solution must satisfy $Da_i^{-n} c_{iT} \to (1+B)^{1\nu}$ as $y_T \to \infty$ at leading order. This condition can be satisfied with $n = 0$, which reverts to $m = 1/2$ and the naive scaling $y_T = Da_i^{1/2}(1-y)$, and to an leading order problem given by

$$\frac{\mathrm{d}^4 c_{iT}^{(0)}}{\mathrm{d}y_T^4} = \frac{\mathrm{d}^2}{\mathrm{d}y_T^2}\left[\left(c_{iT}^{(0)}\right)^\nu\right], \tag{S46a}$$

$$c_{iT}^\nu - \frac{\mathrm{d}^2 c_{iT}}{\mathrm{d}y_T^2} = 0 \text{ at } y_T = 0, \tag{S46b}$$

$$\frac{\mathrm{d}c_{iT}^{(0)}}{\mathrm{d}y_T} = 0 \text{ at } y_T = 0, \tag{S46c}$$

$$c_{iT}^{(0)} \to (1+B)^{1/\nu} \text{ as } y_T \to \infty, \tag{S46d}$$

which only admits a solution if $B = -1$, which contradicts our prior assumption. The only option left is

then that $B = -1$ but $n \neq 0$, in which case the outer solution can be expanded as

$$c_i(y_T) = Da_i^{-m/\nu} y_T^{1/\nu} + O(Da_i^{-1}). \tag{S47}$$

The leading-order matching condition at leading order is then $Da_i^{-n} c_{iT} \to Da_i^{-m/\nu} y_T^{1/\nu}$ as $y_T \to \infty$, which requires $m = \nu n$. In combination with the previous condition of $n = (1 - 2m)/(\nu - 1)$, this leads to

$$m = \frac{\nu}{3\nu - 1}, \tag{S48a}$$

$$n = \frac{1}{3\nu - 1}, \tag{S48b}$$

with values tabulated in Table S1.

Table S1: Values of the exponents m and n in Equation (S48) for different values of ν.

ν	2	3	4	5
m	2/5	3/8	4/11	5/14
n	1/5	1/8	1/11	1/14

With these exponents, we arrive at the zeroth-order inner problem

$$\frac{\mathrm{d}^4 c_{iT}^{(0)}}{\mathrm{d}y_T^4} = \frac{\mathrm{d}^2}{\mathrm{d}y_T^2}\left[\left(c_{iT}^{(0)}\right)^{\nu}\right], \tag{S49a}$$

$$c_{iT}^{\nu} - \frac{\mathrm{d}^2 c_{iT}}{\mathrm{d}y_T^2} = 0 \text{ at } y_T = 0, \tag{S49b}$$

$$\frac{\mathrm{d}c_{iT}^{(0)}}{\mathrm{d}y_T} = 0 \text{ at } y_T = 0, \tag{S49c}$$

$$c_{iT}^{(0)} \to y_T^{1/\nu} \text{ as } y_T \to \infty, \tag{S49d}$$

which can be integrated twice to yield

$$\frac{\mathrm{d}^2 c_{iT}^{(0)}}{\mathrm{d}y_T^2} = \left(c_{iT}^{(0)}\right)^{\nu} - y_T, \tag{S50a}$$

$$\frac{\mathrm{d}c_{iT}^{(0)}}{\mathrm{d}y_T} = 0 \qquad \text{at } y_T = 0, \tag{S50b}$$

$$c_{iT}^{(0)} \to y_T^{1/\nu} \qquad \text{as } y_T \to \infty. \tag{S50c}$$

As opposed to all the cases outlined above, this problem is nonlinear and does not, in general, have an exact solution. In the particular case of $\nu = 2$, the problem can be mapped to the so-called first Painlevé transcendent[S1] after a change of variables. This problem has been shown to lack exact solutions in terms of

elementary functions so, in order to find a local expansion of its solution, we pose a Taylor series

$$c_{iT}^{(0)} = \alpha + \beta y_T^2 + O(y_T^3), \tag{S51}$$

introduce it in (S50), and obtain $\beta = \alpha^\nu/2$, and where the parameter $\alpha = c_{iT}(0)$ must be calculated by numerically solving (S50).

In summary, the solutions near the bottom and top walls can then be expressed as

$$c_{iB} = 1 - \frac{Da_i^{-1/2}}{\nu}\left[y_B + \frac{e^{-\sqrt{\nu}y_B}}{\nu^{1/2}}\right] + O\left(Da_i^{-1}\right), \tag{S52a}$$

$$c_{iT} = \alpha + \frac{\alpha^\nu}{2} y_T^2 + O\left(y_T^3\right). \tag{S52b}$$

Changing coordinates to $y_+ = Da_i^{-1/2} y_B$ and $y_- = Da_i^{-\frac{\nu}{3\nu-1}} y_T$ and rescaling, the expansions of the solution near the walls can be obtained as

$$c_{iB} \approx \left[1 - \frac{Da_i^{-1/2}}{\nu^{3/2}}\right] - Da_i^{1/2}\frac{y_+^2}{2\nu^{1/2}} \quad \text{at the source,} \tag{S53a}$$

$$c_{iT} \approx Da_i^{-\frac{1}{3\nu-1}}\alpha + Da_i^{\frac{2\nu-1}{3\nu-1}}\frac{\alpha^\nu}{2}y_-^2 \qquad \text{at the sink,} \tag{S53b}$$

which coincide with Equations (42) from the main text.

S3. Effect of dissociation chemistry on electric double layers

To elucidate if the chemical reactions given by equation (1) affect the double layer structure, we consider the governing equations for the cation and anion concentration within this region. Since within the double layer electroneutrality does not hold, we consider the Nernst-Planck equation for the cationic $C_\mathcal{C} = [\mathcal{C}]$ and the anionic $C_\mathcal{A} = [\mathcal{A}]$ species separately, and denote them with an index j that can be either $j = \mathcal{C}$ or $j = \mathcal{A}$. The equations are[S2,S3]

$$\boldsymbol{\nabla}\cdot(\boldsymbol{U}C_j) = \boldsymbol{\nabla}\cdot\left[D_j\left(\boldsymbol{\nabla}C_j + \frac{z_j e}{k_B T}C_j\boldsymbol{\nabla}\Psi\right)\right] + \nu_j\left[k_f C_s - k_r C_\mathcal{C}^{\nu_\mathcal{C}} C_\mathcal{A}^{\nu_\mathcal{A}}\right], \tag{S54}$$

with z_j the valence of each species, e the elementary charge, k_B the Boltzmann constant, T the absolute temperature, D_j each of the species diffusivities, $\boldsymbol{U}$ the velocity field, and Ψ the electric potential. The solute concentration C_s, dissociation coefficients ν_j, and rate constants k_f and k_r are introduced in the Theory section of the main text. We then split the operators (resp. the velocity field) into a component Y (resp.

U_Y) perpendicular to the particle wall (assumed to lie at $Y = 0$) and tangential components:

$$\begin{aligned}\boldsymbol{\nabla}_{\parallel}\boldsymbol{\cdot}(\boldsymbol{U}_{\parallel}C_j) + \frac{\partial}{\partial Y}(U_Y C_j) = \boldsymbol{\nabla}_{\parallel}\boldsymbol{\cdot}\left[D_j\left(\boldsymbol{\nabla}_{\parallel}C_j + \frac{z_j e}{k_B T}C_j\boldsymbol{\nabla}_{\parallel}\Psi\right)\right] +& \\ + \frac{\partial}{\partial Y}\left[D_j\left(\frac{\partial C_j}{\partial Y} + \frac{z_j e}{k_B T}C_j\frac{\partial \Psi}{\partial Y}\right)\right] +& \\ + \nu_j\left[k_f C_s - k_r C_{\mathcal{C}}^{\nu_{\mathcal{C}}} C_{\mathcal{A}}^{\nu_{\mathcal{A}}}\right].&\end{aligned} \tag{S55}$$

Next, we nondimensionalize variables using the typical scales in the boundary layer [S3], namely:

$$\begin{aligned}\boldsymbol{\nabla}_{\parallel} = \frac{1}{R_p}\hat{\boldsymbol{\nabla}}_{\parallel}, \qquad Y = \lambda_D y, \qquad \boldsymbol{U}_{\parallel} = \left(\frac{\lambda_D^2 k_B T C_{i0}}{\mu R_p}\right)\boldsymbol{u}_{\parallel}, \qquad U_Y = \frac{\lambda_D}{R_p}\left(\frac{\lambda_D^2 k_B T C_{i0}}{\mu R_p}\right)u_y, \\ \Psi = \left(\frac{k_B T}{e}\right)\psi, \qquad C_j = \nu_j C_{i0} c_j, \qquad C_s = C_{s0} c_s,\end{aligned} \tag{S56}$$

with $\lambda_D = \sqrt{\epsilon k_B T/(e^2 C_{i0})}$ the Debye length, ϵ the medium permittivity, R_p the particle radius, μ the fluid viscosity, and C_{s0} and C_{i0} the scales for solute and ionic concentration (respectively) defined in the main text. Note that, following the main text, all dimensionless variables are lowercase. Using the fact that $k_r \nu_{\mathcal{C}}^{\nu_{\mathcal{C}}} \nu_{\mathcal{A}}^{\nu_{\mathcal{A}}} C_{i0}^{(\nu_{\mathcal{C}}+\nu_{\mathcal{A}})} = k_f C_{s0}$ from chemical equilibirum and rearranging, we finally obtain

$$\begin{aligned}\left(\frac{\lambda_D}{R_p}\right)^2 Pe_j^{DL}\left[\hat{\boldsymbol{\nabla}}_{\parallel}\cdot(\boldsymbol{u}_{\parallel}c_j) + \frac{\partial}{\partial y}(U_y c_j)\right] = \left(\frac{\lambda_D}{R_p}\right)^2\left[\hat{\boldsymbol{\nabla}}_{\parallel}\cdot\left(\hat{\boldsymbol{\nabla}}_{\parallel}c_j + z_j c_j\hat{\boldsymbol{\nabla}}_{\parallel}\psi\right)\right] +& \\ + \frac{\partial}{\partial y}\left[\left(\frac{\partial c_j}{\partial y} + z_j c_j\frac{\partial \psi}{\partial y}\right)\right] +& \\ + \left(\frac{\lambda_D}{R_p}\right)^2 Da_j^{DL}\left[c_s - c_{\mathcal{C}}^{\nu_{\mathcal{C}}} c_{\mathcal{A}}^{\nu_{\mathcal{A}}}\right].&\end{aligned} \tag{S57}$$

where we have defined the double-layer Péclet and Damköhler numbers

$$Pe_j^{DL} = \frac{\epsilon {k_B}^2 T^2}{\mu e^2 D_j}, \qquad Da_j^{DL} = \frac{k_f R_p^2 C_{s0}}{D_j C_{i0}}. \tag{S58}$$

In the limit of thin double layers compared to the particle radius ($\lambda_D \ll R_p$) and moderate values $Pe_j^{DL} \lesssim 1$ and $Da_j^{DL} \lesssim 1$, the leading-order dominant balance of Equation (S57) is purely between wall-normal ion diffusion and electromigration, which leads to the well-known Boltzmann distributions for each of the ionic species, i.e. $C_j = C_{i0}\exp\left[-z_j(\Psi - \Psi_\infty)\right]$, with Ψ_∞ a far-field reference potential.

However, this picture can be altered for high enough values of either Pe_j^{DL} or Da_j^{DL}. Specifically, chemical reactions can alter the ion distributions if $(\lambda_D/R_p)^2 Da_j^{DL} = O(1)$. Noting that $Da_j^{DL} = (R_p/W)^2(D_i/D_j)Da_i$, where W is the channel width, D_i the electrolyte ambipolar diffusivity, and Da_i the ionic Damköhler number defined in Equation (21b) of the main text, we can ensure that chemical reactions are negligible in the double

layer structure only if

$$\left(\frac{\lambda_D}{W}\right)^2 \frac{D_i}{D_j} Da_i \ll 1 \tag{S59}$$

and, since we can assume the ambipolar diffusivity D_i to be of the same order of magnitude as D_j, we can simplify the expression into

$$Da_i \ll \left(\frac{W}{\lambda_D}\right)^2, \tag{S60}$$

which is the same as Equation (48) of the main text.

S4. Outline of experimental parameters

Table S2 summarizes the values of constants assumed in the experiments, as well as the parameters estimated from these constants.

S5. Calibration of particle fluorescence intensity

In order to corroborate that the measured fluorescence intensity is proportional to the particle concentration, we run calibration tests in which particle suspensions at different volume fractions are driven through the same experimental setup, but without the injection of CO_2 or N_2 gases. This causes the particle distribution to remain homogeneous with the known volume fraction introduced through the inlet. Plots of the fluorescent signal as a function of the volume fraction are presented in Figure S1.

References

(S1) Bender, C. M.; Orszag, S. A. *Advanced mathematical methods for scientists and engineers I: Asymptotic methods and perturbation theory*; Springer, New York, NY, 1999.

(S2) Prieve, D. C.; Anderson, J. L.; Ebel, J. P.; Lowell, M. E. Motion of a particle generated by chemical gradients. Part 2. Electrolytes. *J. Fluid Mech.* **1984**, *148*, 247–269.

(S3) Gupta, A.; Rallabandi, B.; Stone, H. A. Diffusiophoretic and diffusioosmotic velocities for mixtures of valence-asymmetric electrolytes. *Phys. Rev. Fluids* **2019**, *4*, 043702.

(S4) Merkel, T. C.; Bondar, V. I.; Nagai, K.; Freeman, B. D.; Pinnau, I. Gas sorption, diffusion, and permeation in poly(dimethylsiloxane). *J. Polym. Sci. B Polym. Phys.* **2000**, *38*, 415–434.

(S5) Haynes, W. M. *CRC Handbook of Chemistry and Physics*; CRC Press, 2014.

Table S2: Constants and estimated parameters in the experiments.

Parameter	Symbol	Value	Units	Info
PDMS permeability	$\mathcal{P}_{\mathrm{PDMS}}$	1.270×10^{-12}	$\mathrm{mol\,(m\,s\,Pa)^{-1}}$	Ref. S4
PDMS wall width	w	1.000×10^{-4}	m	
Main channel length	L	5.500×10^{-2}	m	
Entry channel length	ℓ	6.000×10^{-3}	m	
Atmospheric pressure	p_{atm}	1.013×10^{5}	Pa	
Applied CO_2 pressure	$p_{\mathrm{CO_2}}$	1.380×10^{4}	Pa	
Water viscosity	μ	1.000×10^{-3}	$\mathrm{kg\,(m\,s)^{-1}}$	
Water permittivity	ϵ	6.940×10^{-10}	$\mathrm{C\,(V\,m)^{-1}}$	
Boltzmann constant	k_{B}	1.381×10^{-23}	$\mathrm{J\,K^{-1}}$	
Avogadro number	N_{A}	6.022×10^{23}	$\mathrm{mol^{-1}}$	
Absolute temperature	T	298	K	
Elementary charge	e	1.602×10^{-19}	C	
Solute diffusivity	D_s	1.910×10^{-9}	$\mathrm{m^2\,s^{-1}}$	Ref. S5
Cation diffusivity	$D_{\mathcal{C}}$	9.311×10^{-9}	$\mathrm{m^2\,s^{-1}}$	Ref. S5
Anion diffusivity	$D_{\mathcal{A}}$	1.185×10^{-9}	$\mathrm{m^2\,s^{-1}}$	Ref. S5
Forward reaction rate	k_f	3.900×10^{-2}	$\mathrm{s^{-1}}$	Ref. S6
Reverse reaction rate	k_r	9.200×10^{1}	$\mathrm{m^3\,(mol\,s)^{-1}}$	Ref. S6
CO_2 Henry constant	K_{H}	2.980×10^{3}	$\mathrm{Pa\,m^3\,mol^{-1}}$	Ref. S6
Ambipolar diffusivity	D_i	2.102×10^{-9}	$\mathrm{m^2\,s^{-1}}$	$D_i = 2D_{\mathcal{C}}D_{\mathcal{A}}/(D_{\mathcal{C}} + D_{\mathcal{A}})$
Electrophoresis parameter	β	7.742×10^{-1}	–	$\beta = (D_{\mathcal{C}} - D_{\mathcal{A}})/(D_{\mathcal{C}} + D_{\mathcal{A}})$
CO_2 pressure at measurement	p_0	1.026×10^{5}	Pa	$p_0 = \sqrt{p_{\mathrm{atm}}^2 + \frac{\ell\,(p_{\mathrm{CO_2}}^2 - p_{\mathrm{atm}}^2)}{L + 2\ell}}$
Wall mass transfer resistance	$\mathcal{R}_w$	7.874×10^{7}	$\mathrm{m^2 s\,Pa\,mol^{-1}}$	$\mathcal{R}_w = w/\mathcal{P}_{\mathrm{PDMS}}$
Channel mass transfer resistance	$\mathcal{R}_c$	3.900×10^{8}	$\mathrm{m^2 s\,Pa\,mol^{-1}}$	$\mathcal{R}_c = WK_{\mathrm{H}}/D_s$
Characteristic solute concentration	C_{s0}	2.948×10^{1}	mM	$C_{s0} = \frac{\mathcal{R}_c + \mathcal{R}_w}{\mathcal{R}_c + 2\mathcal{R}_w}\frac{p_0}{K_{\mathrm{H}}}$
Characteristic ionic concentration	C_{i0}	1.118×10^{-1}	mM	Equation (9a)
Debye length	λ_D	2.874×10^{-8}	m	$\lambda_D = \sqrt{\frac{\epsilon k_B T}{e^2 C_{i0}}}$
Solute Damköhler number	Da_s	1.276	–	Equation (21a)
Ionic Damköhler number	Da_i	3.057×10^{2}	–	Equation (21b)
Particle mobility	Γ_p	Variable	$\mathrm{m^2 s^{-1}}$	From S7, CC model
Particle Brownian diffusivity	D_p	Variable	$\mathrm{m^2 s^{-1}}$	$D_p = k_B T/(6\pi\mu R_p)$

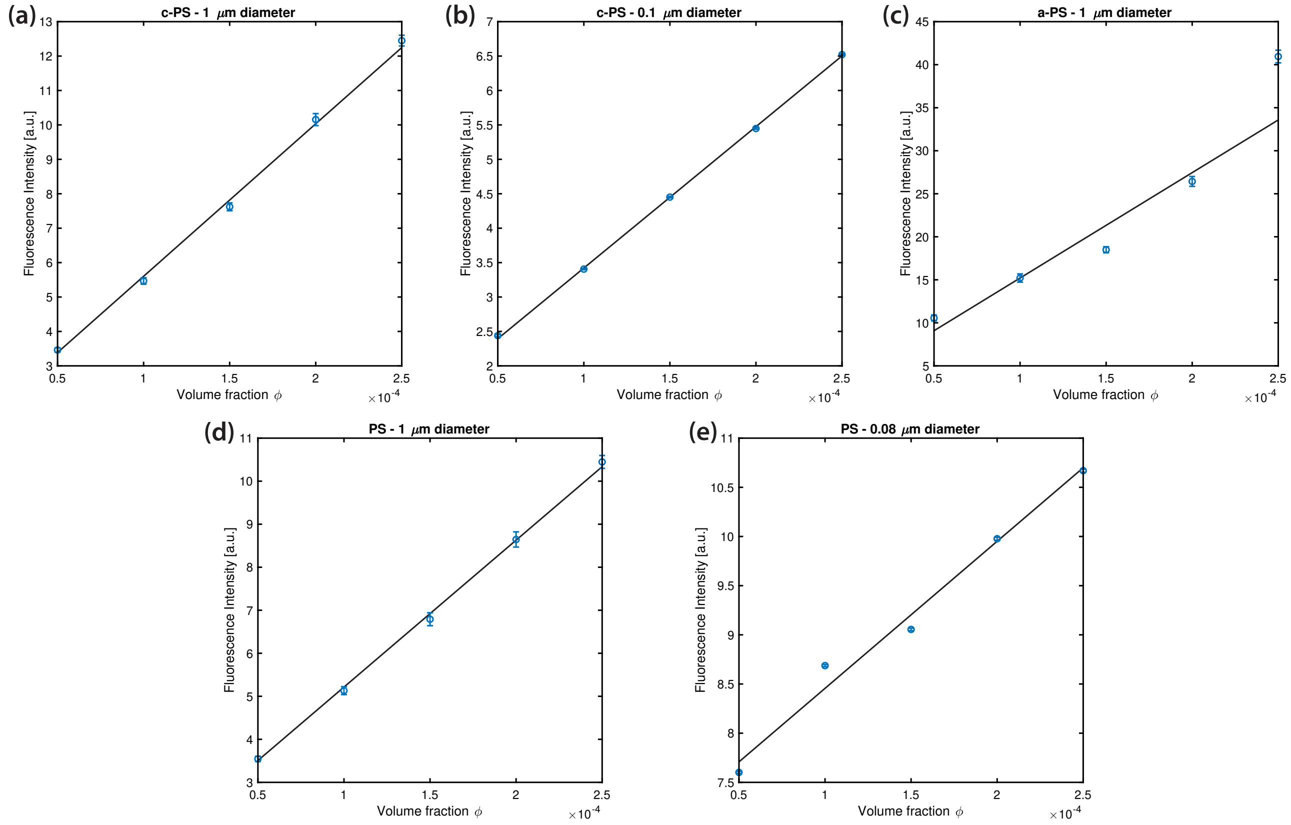

Figure S1: Fluorescence calibration tests for five particle types and sizes. The error bars indicate the standard deviation resulting from averaging the fluorescence signal in time, X, and Y for each calibration test. The black line corresponds to a linear least-squares fit weighted by the standard deviations.

(S6) Shin, S.; Shardt, O.; Warren, P. B.; Stone, H. A. Membraneless water filtration using CO_2. *Nat. Commun.* **2017**, *8*, 15181.

(S7) Gupta, A.; Shim, S.; Stone, H. A. Diffusiophoresis: from dilute to concentrated electrolytes. *Soft Matter* **2020**, *16*, 6975–6984.
